# Spinal manipulation characteristics: a scoping literature review of force-time characteristics

**DOI:** 10.1186/s12998-023-00512-1

**Published:** 2023-09-13

**Authors:** Lindsay M Gorrell, Luana Nyirö, Mégane Pasquier, Isabelle Pagé, Nicola R Heneghan, Petra Schweinhardt, Martin Descarreaux

**Affiliations:** 1 Integrative Spinal Research Group, Department of Chiropractic Medicine, University Hospital Balgrist and University of Zurich, Zurich, Switzerland; 2 Department of Anatomy, Université du Québec à Trois-Rivières, Trois-Rivières, QC Canada; 3 Institut Franco-Européen de Chiropraxie, Toulouse, France; 4 Department of Chiropractic, Université du Québec à Trois-Rivières, Trois-Rivières, QC Canada; 5 Center for Interdisciplinary Research in Rehabilitation and Social Integration (Cirris), Centre Intégré Universitaire de Santé et de Services Sociaux de la Capitale-Nationale (CIUSSS-CN), Québec City, QC Canada; 6 School of Sport, Exercise & Rehabilitation Sciences, University of Birmingham, Birmingham, UK; 7 Department of Human Kinetics, Université du Québec à Trois-Rivières, Trois-Rivières, QC Canada

**Keywords:** Spinal manipulation, Biomechanics, Force-time characteristics, Kinetics, Kinematics, Spine Pain

## Abstract

**Background:**

Spinal manipulation (SM) is a recommended and effective treatment for musculoskeletal disorders. Biomechanical (kinetic) parameters (e.g. preload/peak force, rate of force application and thrust duration) can be measured during SM, quantifying the intervention. Understanding these force-time characteristics is the first step towards identifying possible active ingredient/s responsible for the clinical effectiveness of SM. Few studies have quantified SM force-time characteristics and with considerable heterogeneity evident, interpretation of findings is difficult. The aim of this study was to synthesise the literature describing force-time characteristics of manual SM.

**Methods:**

This scoping literature review is reported following the Preferred Reporting Items for Scoping Reviews (PRISMA-ScR) statement. Databases were searched from inception to October 2022: MEDLINE (Ovid), Embase, CINAHL, ICL, PEDro and Cochrane Library. The following search terms and their derivatives were adapted for each platform: spine, spinal, manipulation, mobilization or mobilisation, musculoskeletal, chiropractic, osteopathy, physiotherapy, naprapathy, force, motor skill, biomechanics, dosage, dose-response, education, performance, psychomotor, back, neck, spine, thoracic, lumbar, pelvic, cervical and sacral. Data were extracted and reported descriptively for the following domains: general study characteristics, number of and characteristics of individuals who delivered/received SM, region treated, equipment used and force-time characteristics of SM.

**Results:**

Of 7,607 records identified, 66 (0.9%) fulfilled the eligibility criteria and were included in the analysis. Of these, SM was delivered to the cervical spine in 12 (18.2%), the thoracic spine in 40 (60.6%) and the lumbopelvic spine in 19 (28.8%) studies. In 6 (9.1%) studies, the spinal region was not specified. For SM applied to all spinal regions, force-time characteristics were: preload force (range: 0-671N); peak force (17-1213N); rate of force application (202-8700N/s); time to peak thrust force (12-938ms); and thrust duration (36-2876ms).

**Conclusions:**

Considerable variability in the reported kinetic force-time characteristics of SM exists. Some of this variability is likely due to differences in SM delivery (e.g. different clinicians) and the measurement equipment used to quantify force-time characteristics. However, improved reporting in certain key areas could facilitate more sophisticated syntheses of force-time characteristics data in the future. Such syntheses could provide the foundation upon which dose-response estimates regarding the clinical effectiveness of SM are made.

**Supplementary Information:**

The online version contains supplementary material available at 10.1186/s12998-023-00512-1.

## Introduction

The prevalence of musculoskeletal disorders, including low back and neck pain, is increasing globally [1, 2]. Based on age-standardized disability-adjusted life years, musculoskeletal disorders are currently ranked the 5th highest globally compared to a ranking of 10th in 1990 [3, 4]. In 2017, low back pain was the most prevalent musculoskeletal disorder globally (36.8%), with neck pain the third most prevalent (18.4%). These disorders are not only disabling but also costly, with low back and neck pain having the highest amount of health care spending by payor in the United States (US$134.5 billion in 2016) [5]. Conservative treatments (e.g. spinal manipulation (SM)) are recommended and effective treatments for musculoskeletal disorders as part of multimodal therapy [6–9]. SM is characterized by a single high-velocity, low-amplitude (HVLA) thrust delivered to a joint with the intention of moving the articulation past its physiological range of motion but without exceeding its anatomic limit [10]. Force-time characteristics such as preload and peak force, rate of force application and thrust duration can be measured during the application of SM, allowing for biomechanical quantification of the intervention. Understanding these force-time characteristics is the first step towards identifying possible active ingredient/s responsible for the clinical effectiveness (e.g. decreased pain and increased range of motion (ROM) of the intervention). However, only a few studies quantify the delivery of SM and those that do, do so heterogeneously. Therefore, interpretation of reported results in this area is difficult. Highlighting this, to our best knowledge, there has been only one attempt to synthesise the literature reporting on the force-time characteristics of SM delivered to all regions of the spine [11]. In this 2010 systematic review, preload and peak forces delivered during SM were collated from 15 studies (cervical: n = 4; thoracic: n = 8; and lumbopelvic: n = 3). Downie and colleagues concluded that heterogeneity in the included studies precluded a standardized biomechanical description of HVLA SM but that a relationship between preload, peak force and thrust duration was present. The authors recommended improved reporting of SM force-time characteristics when assessing the clinical efficacy of HVLA SM (e.g. in clinical trials). More recently, Gyer and colleagues performed a critical literature review of 20 studies (humans: n = 12) in which SM was delivered to the thoracic (n = 5), lumbar (n = 6) or, both regions (n = 1) [12]. While force-time characteristics of SM (e.g. thrust force and duration) were reported, the authors primarily investigated the relationship between force-time characteristics of SM and physiological and clinical outcomes. In summary, it was reported that there exists a dose-response relationship between force-time characteristics of SM and transient physiological outcomes (e.g. electromyographical responses); however, it remains unknown what effect varying force-time characteristics of SM might have on clinical outcomes.

As such, improved reporting would provide a starting point for quantification of minimum thresholds (or dosages), for a range of force-time characteristics (e.g. thrust force and duration) and ultimately, for the determination of how these parameters affect the clinical effectiveness of SM. To date, such thresholds have been hypothesised but not systematically investigated [13, 14]. Indeed, similar findings were reported in an earlier scoping review on SM frequency and dosage effects on clinical and physiological outcomes which concluded that dosage effects clearly influence short-term physiological responses to manipulation but found no relationship between the force-time characteristics of SM delivery and clinical outcomes such as decreased pain and/or increased range of motion [15]. It is possible that heterogeneity in the literature (as reported by Downie and colleagues [11]) could be responsible for the observed lack of relationship between SM force-time characteristics and clinical outcomes. Additionally, the existence of a threshold above which the nervous system is sufficiently stimulated to realise a favourable clinical outcome could partially explain why clinical improvements have been reported in studies using different therapeutic approaches [16–18]. Therefore, the aim of this study was to synthesise the existing literature describing biomechanical (kinetic) parameters in the delivery of manual SM.

## Methodology

This scoping literature review was conducted in 5 stages as outlined by Arksey and O’Malley [19]. Specifically: (i) the research question was identified; (ii) potentially relevant studies were identified; (iii) relevant studies were selected; (iv) data were charted; and (v) results were generated by collating, summarizing and reporting the data. The final step (optional consultation process) was not included as it was deemed to be unnecessary in the context of the current study. The Preferred Reporting Items for Scoping Reviews (PRISMA-ScR) statement was used to report the data [20]. The protocol was designed by an international, interprofessional team of chiropractors and physiotherapists with relevant methodological and clinical expertise and registered at the Open Science Framework Registry (https://osf.io/3mqjs/). Protocol deviations included that this study was originally designed (and the searches conducted) to capture information concerning the force-time characteristics of both SM and spinal mobilization (SMob). However, due to the large quantity of data published on this topic, it was decided to report the force-time characteristics of SM and SMob separately. Secondly, it was decided to exclude studies reporting on SM delivered to animals as it was unknown how comparable (biomechanically) the delivery of the intervention was to SM delivered to humans.

### Eligibility criteria

Eligibility criteria were selected by the research team using the Sample, Phenomenon of Interest, Design, Evaluation, Research Type (SPIDER) search concept tool [21].

### Inclusion criteria

S – the sample population was humans (of any age) and inanimate objects (e.g. instrumented tool, manikin);

PI – the phenomenon of interest was manually delivered SM, delivered by any regulated health professional (e.g. chiropractor or physiotherapist) or student enrolled at an accredited institution;

D – observational study designs (e.g. case series studies, cohort and case-control studies);

E – kinetic variables of the intervention (e.g. force-time characteristics); and

R – original quantitative research data from studies utilizing SM as either the sole intervention or as a comparator.

### Exclusion criteria

The following exclusion criteria were used: (i) SM and/or SMob delivered by a mechanical instrument or device; (ii) all other therapeutic modalities; (iii) manuscript not published in English, French or German; and (iv) studies that had been retracted, were secondary analyses, trial registrations, protocols, clinical practice guidelines, commentaries, editorials, conference proceedings or single case studies.

### Search strategy

The following databases were searched from inception to 4 October 2022: MEDLINE(Ovid), Embase, CINAHL, ICL, PEDro and Cochrane Library. Reference lists of included studies were screened to insure all relevant literature was captured. The search strategy was informed by subject specific and methodological experts. The following search terms and derivatives were adapted for each search engine: (spine, spinal, manipulation, mobilization or mobilisation, musculoskeletal, chiropractic, osteopathy, physiotherapy, naprapathy, force, motor skill, biomechanics, dosage, dose-response, education, performance, psychomotor, back, neck, spine, thoracic, lumbar, pelvic, cervical and sacral). Search strategies for all databases are provided in Appendix 1.

### Study selection process

Records retrieved from the electronic searches were exported to the Rayyan© online platform (2022) [22] and duplicate records were removed. Groups of two authors (LG and LN; LG and IP; LG and MP) independently screened potentially eligible studies in a step-wise process, beginning with review of each title and abstract. Full-texts of the studies remaining after the first phase of screening were retrieved and further screened against the eligibility criteria by groups of two authors (LG and LN; LG and IP). Any disagreements regarding inclusion were resolved by consensus and if consensus could not be reached, disagreements were resolved by a third author (MD).

### Data extraction

Data were extracted from eligible studies by groups of two authors (LG and LN; LG and MP). These data included: (i) general study characteristics (e.g. title, author, year and country of publication and type of study); (ii) general study information (e.g. individual who delivered the intervention [e.g. clinician, student], professional qualification of individual delivering the intervention [e.g. chiropractor, physiotherapist], years of clinician experience/number of student hours, number of clinicians/students who delivered SM or SMob, recipient [e.g. human, manikin], number of recipients, whether the intervention was SM or SMob [and grade of mobilization], the region treated [e.g. cervical, thoracic] and the measurement equipment used to record force-time characteristics of the intervention); and (iii) force-time characteristics of SM (e.g. preload and peak forces, rate of force application). Data reporting on SMob will be published elsewhere (manuscript in preparation). Given the focus on describing and detailing studies that fulfilled eligibility criteria to enable the study aim to be fulfilled, no assessment of study quality was performed.

### Data synthesis

Data are reported using descriptive statistics (mean, standard deviation and range) where possible. Deviations to this are indicated in the tables (e.g. 95% confidence intervals or median and interquartile range) and reflect how the data were reported in the original studies. Frequencies and proportions of trials reporting on each of the specified domains above were calculated in Microsoft Excel (Office 365, Microsoft Corporation, USA).

To streamline the large amount of data reported here, the following decisions were made regarding how to best report the data and are indicated in the tables: (i) for studies reporting forces measured in 3-dimensions (3D) and including the resultant forces (i.e. the total forces applied), only the resultant forces are reported; and (ii) for studies measuring forces applied in 3D but not including the resultant forces, only the forces measured in the primary direction of applied force are reported in the tables (e.g. for prone posterior-anterior thoracic SM, the vertical forces are reported). Regarding the reporting of metrological data of the equipment used to measure the force-time characteristics, a consensus was reached by two authors (LG and MD) as to whether adequate information was provided. In cases where metrological details were reported (e.g. it was stated that calibration of equipment was performed and/or values for equipment accuracy were provided) but no further information was given and/or it was not possible to know how these values were obtained, this was recorded as metrological data were not provided. Considering definitions used in the literature to describe the duration of applied SM, considerable variability was observed. For example, the reporting of time to peak thrust force (i.e. from the end of preload to the peak force of the thrust) was often described as thrust duration [23], yet in other instances, both time to peak thrust force and thrust duration (i.e. the duration of force application (e.g. [24]) were reported. To ensure the correct reporting of this data, the following steps were taken to determine which domain was reported: (i) when the definition was provided in the manuscript, this was used; (ii) if the definition was not provided but figure/s and/or graph/s were provided, these were used; and (iii) if the definition and figure/s and/or graphs were not provided, the terminology used by the original authors was kept. This decision was achieved by consensus of the two independent data extractors (LG and LN; LG and MP).

## Results

**Fig. 1:**
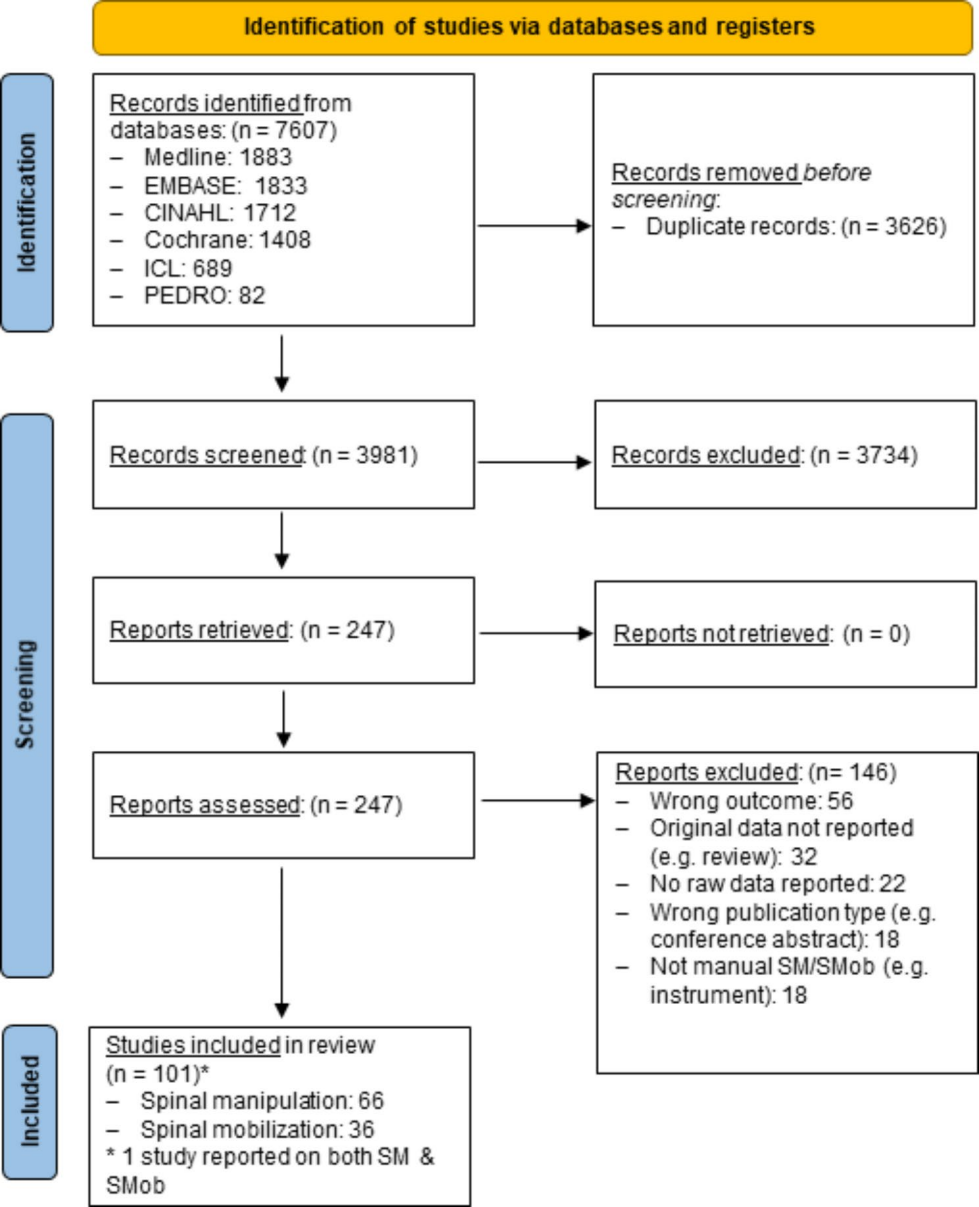
PRISMA flow diagram

There were 7,607 records initially identified by the electronic searches (Fig. 1). A total of 3,981 unique records remained after de-duplication (n = 3,626). After title and abstract screening, full texts of the 247 remaining reports were screened. Of these, 66 reported on SM, fulfilled the eligibility criteria and were included in the analysis. The reference list for these studies is provided in Appendix 2 and the reference number provided in each of the tables relates to the numbering in this Appendix. The most common reasons for exclusion were: the paper reported on the wrong outcome (e.g. did not report on the force time parameters of SM (n = 56)) and original data were not reported (e.g. the paper was a review (n = 32)).

Of the 66 included studies, 30 (45.5%) were published in the previous 10 years (Table 1). Most studies were conducted in Canada (n = 28, 42.4%), followed by the USA (n = 23, 34.8%). Typically, the study design was cross-sectional (n = 54, 81.8%), with SM delivered by clinicians only (i.e. no students were involved) (n = 43, 65.2%), whose profession was chiropractic (n = 57, 86.4%). In the 54 (81.8%) studies in which SM was delivered by clinicians, clinicians with more than 5 years of experience were most commonly involved (n = 26, 47.3%). However, clinician experience was not reported in 18 (32.7%) studies. When SM was delivered by a student (n = 23, 34.8%), the number of HVLA manual SM training hours was not reported in 20 (87.0%) studies. In most studies, the number of individuals (i.e. clinicians and/or students) delivering SM was between 1–49 (n = 50, 75.8%), with only 1–2 individuals delivering SM in 27 (54.0%) studies. SM was delivered to adults (18–65 years) in 27 (40.9%) studies, with the characteristics of the participants to which SM was delivered not reported in 11 (16.7%) studies. The number of individuals receiving SM was reported as between 1–49 in 59 (89.4%) studies, with only 1–2 individuals receiving SM in 28 (47.5%) studies. SM was most commonly delivered to the thoracic spine (n = 40, 60.6%) and the lumbopelvic spine (n = 19, 28.8%). The SM ‘technique’ (e.g. ‘toggle’, ‘Diversified’, ‘Gonstead’) was reported in 63 (95.5%) studies. Force-time characteristics were measured at the clinician-patient interface in 23 (34.8%) studies, the patient-table interface in 21 (31.8%) studies, both interfaces in 6 (9.1%) studies and was not reported in 16 (24.2%) studies. Metrological data of the measurement equipment were not reported in 53 (80.3%) studies. Regarding force-time characteristics, the following were reported: preload force in 42 (63.6%) studies; peak force in 57 (86.4%) studies; rate of force application in 34 (51.5%) studies; time from end of preload force to peak force of thrust in 36 (54.5%) studies; and thrust duration in 21 (31.8%) studies.

**Table 1:** Overall summary of studies reporting on the force-time characteristics of spinal manipulation (SM) (n = 66)

	n (%)			n (%)
**Year, n = 66**			**Who received SM, n = 66**	
2013 to 2022	30 (45.5)		Adult (18 to 65y)	27 (40.9)
2003 to 2012	18 (27.3)		Geriatric (> 65y)	1 (1.5)
1993 to 2002	18 (27.3)		Cadaver	2 (3.0)
**Country, n = 66**			Instrumented tool/force plate	7 (11.0)
Australia	2 (3.0)		Mannikin	16 (24.2)
Belgium	2 (3.0)		Mixed	2 (3.0)
Canada	28 (42.4)		Unclear	11 (16.7)
China	1 (1.5)		**Number of individuals receiving SM, n = 66**	
England	3 (4.5)		1 or 2	28 (42.4)
France	4 (6.1)		1 to 49	59 (89.4)
Italy	2 (3.0)		50 to 99	5 (7.6)
Korea	1 (1.5)		100 to 149	1 (1.5)
Spain	1 (1.5)		Not reported	1 (1.5)
Unclear	1 (1.5)		Region SM delivered to, n = 66*	
USA	23 (34.8)		Cervical	12 (18.2)
**Study type, n = 66**			Thoracic	40 (60.6)
Cross-sectional	54 (81.8)		Lumbopelvic	19 (28.8)
Prospective	9 (13.6)		Not specified	6 (9.1)
Both	1 (1.5)		**Technique reported, n = 66**	
Unclear	2 (3.0)		Yes	63 (95.5)
**Individual who delivered SM, n = 66**			No	3 (4.5)
Practitioner	43 (65.2)		**Measurement interface, n = 66**	
Student	11 (16.7)		Clinician-patient	23 (34.8)
Both	11 (16.7)		Patient-table	21 (31.8)
Unclear	1 (1.5)		Both	6 (9.1)
**Profession, n = 66**			Other/not reported	16 (24.2)
Chiropractor	57 (86.4)		**Metrological data reported, n = 66**	
Medical Doctor	1 (1.5)		Reported	13 (19.7)
Physiotherapist	4 (6.1)		Not reported	53 (80.3)
Other/not reported	4 (6.1)		**Preload force, n = 66**	
**Experience (clinician) n = 55**			Reported	42 (63.6)
> 5yr	26 (47.3)		Not reported	24 (36.4)
Mixed	11 (20.0)		**Peak force, n = 66**	
Unclear	18 (32.7)		Reported	57 (86.4)
**Hours of training (student) n = 23**			Not reported	9 (13.6)
Reported	3 (13.0)		**Rate of force application, n = 66**	
Not reported	20 (87.0)		Reported	34 (51.5)
**Number of individuals delivering SM, n = 66**			Not reported	32 (48.5)
1 or 2	27 (40.9)		**Time to peak, n = 66**	
1 to 49	50 (75.8)		Reported	36 (54.5)
50 to 99	7 (10.6)		Not reported	30 (45.5)
100 to 149	7 (10.6)		**Thrust duration, n = 66**	
Not reported	2 (3.0)		Reported	21 (31.8)
			Not reported	45 (68.2)

Abbreviations: n: number of studies, SM: spinal manipulation, USA: United States of America, y: years, >: greater than, *: sums to > 100% as some studies reported on SM delivered to multiple spinal regions

**Table 2:** Summary of studies reporting on the force-time characteristics of spinal manipulation (SM) delivered to the cervical spine of humans (n = 9) and inanimate objects (e.g. human analogue manikins, instrumented tools) (n = 3)

Author/s Year, Country	SM delivery Profession (n)	Experience	Recipient/s (n)	Location/s	Technique/s	Interface/s	Measurement equipment	Metrological data
**Humans**								
Kawchuk et al. 1992, Canada^40^	Clin Chiro (2)	> 5y	NR (2)	C1/C2	Toggle	Clin-pat	Force pad	No
Herzog et al. 1993, Canada^35^	Clin Chiro (60)	NR	NR (58)	Cervical	Lateral-medial	Clin-pat	Force pad	No
Kawchuk et al. 1993, Canada^41^	Clin Chiro (5)	NR	NR (NR)	NR	Lateral break/ Gonstead/Toggle/Rotation	Clin-pat	Force pad	No
Van Zoest et al. 2003, England^65^	Clin Chiro (2)	> 5y	Adult (10)	Mid cervical	Diversified	Clin-pat	Force sensor	Yes
Symons et al. 2012, Canada^57^	Clin Chiro (2)	> 5y	Mixed (33) *Living: 28* *Cadavers: 5*	Living: MP Cadavers: C2-3/ C4-5	Diversified	Clin-pat	Pressure pad	No
Anderst et al. 2018, USA^4^	Clin Chiro (1)	> 5y	Adult (5)	C3/C4/C5	Pillar push	Clin-pat	Pressure pad	No
Gorrell et al. 2020, Canada^29^	Clin Chiro (1)	> 5y	Adult (27)	C1/C2/C6/ C7	Diversified	Clin-pat	Pressure pad	No
Duquette et al. 2021, Canada^23^	Stud Chiro (76)	NR (4th y)	Mixed ages (76)	NR	Lateral index/ pillar push	Pat-table	Force plate	No
Chang et al. 2022, China^10^	Clin MD (1)	> 5y	Adult (34)	C5	Seated resisted rotation	Clin-pat	Mechanical measurement system	No
**Inanimate objects**								
Graham et al. 2010, Australia^30^	Clin & Stud Chiro (13)	Clin: >5y Stud: NR (5th y)	Load cell (1)	NR	Toggle-recoil	Clin-tool	Load cell	No
Triano et al. 2017, Canada^64^	Clin Chiro (1)	> 5y	Manikin (1)	Cervical	Supine rotational	Clin-man & Man-table	Load cell & Force plate	Yes
Duquette et al. 2021, Canada^23^	Stud Chiro (76)	NR (4th y)	Manikin (1)	NR	Lateral index/ pillar push	Man-table	Force plate	No

All superscript numbers in the first column refer to Appendix 2. Abbreviations: C: cervical, Chiro: chiropractor, Clin: clinician, Man: manikin, MD: medical doctor, Mixed: experience of clinicians both > and < 5 years, MP: most painful level, (n): number of participants, NR: not reported, Pat: patient, SM: spinal manipulation, Stud: students, y: years, >: greater than

**Table 3:** Summary of force-time characteristics reported by region for studies reporting on spinal manipulation (SM) (n = 66)

	Location of measurement n (%)	Metrologic data reported n (%)	Preload force reported range (N)	Peak force reported range (N)	Rate of force application reported range (N/s)	Time to peak reported range (ms)	Thrust duration reported range (ms)
Cervical spine (n = 12)							
Humans (n = 9)	Clinician-patient: 8 (88.9)	1 (11.1)	0-162	41–407	440–1787	30–195	90–130
	Patient-table: 1 (11.1)	0	10–13	47–49	NR	NR	NR
Inanimate objects (n = 3)	Clinician-tool: 1 (33.3)	0	0–5	18–246	NR	20–100	NR
	Man-table: 1 (33.3)	0	19–23	123–126	NR	NR	NR
	Both: 1 (33.3)	1 (33.3)	NR	20–112	NR	NR	NR
Thoracic spine (n = 40)							
Humans (n = 27)	Clinician-patient: 11 (40.7)	1 (3.7)	0-254	212–573	416–7000	108–541	318–1330
	Patient-table: 9 (33.3)	5 (18.5)	41–671	290–878	1769–8700	91–280	120–310
	Both: 4 (14.8) Clinician-patient Patient-table	3 (11.1)	260–273 217–310	470–1213 463–1044	1645–3109 1639–15,592*	165–181 96–170	NR NR
	Clinician-ground: 2 (7.4)	0	338–399	564–658	NR	NR	NR
	Accelerometers: 1 (3.7)	0	NR	NR	NR	NR	61
Inanimate objects (n = 13)	Man-table: 5 (38.5)	3 (23.1)	137–172	337–536	2381–3490	109–137	87–198
	Within man: 1 (7.7)	0	105–133	287–304	1473–2495	82–132	NR
	Within man & clinician-ground: 4 (30.8)	0	31–177	404–660	2557–4487	101–266	NR
	Clinician-man & man-table: 1 (7.7)	1 (7.7)	NR	17–393	NR	NR	NR
	NR: 2 (15.4)	0	13–254	212–563	416–3780	109–541	318–1330
Lumbopelvic spine (n = 19)							
Humans (n = 12)	Clinician-patient: 5 (41.7)	1 (8.3)	20–190	106–550	202–1621	164–938	200–2876
	Patient-table: 4 (33.3)	0	NR	128–516	630–3813	NR	320–440
	Both: 1 (8.3)	0	NR	242–940	NR	243	NR
	Clinician-patient & clinician-ground: 1 (8.3)	0	106	328	1078	261	770
	Accelerometers: 1 (8.3)	0	NR	NR	NR	NR	139
Inanimate objects (n = 7)	Clinician-man/tool: 3 (42.9)	0	94.4	433	2692	154	41–574
	Man-table: 2 (28.6)	0	95–163	324–714	2450–4640	142–176	NR
	Both: 1 (14.3)	1 (14.3)	NR	18–387	NR	NR	NR
	Clinician-man & clinician-man 1 (14.3)	0	NR	NR	NR	NR	371–441
No region reported (n = 6)							
Inanimate objects (n = 6)	Clinician-tool: 6 (100.0)	0	9–77	46–387	551–1692^§^	12–251	36–98

Note: in instances where only one value is reported, data were only reported by one study. Abbreviations: Both: clinician-patient/equipment & patient/equipment-table, equip: equipment, man: manikin, ms: milliseconds, n: number of studies, N: Newtons, NR: not reported, s: seconds, tool: something to which SM was delivered (e.g. load cell), *: 15,592 N/s reported, likely a mistake [29], §: this is reported as N/ms, likely a mistake [30]

### Cervical spine

Of the 66 included studies, 12 (18.2%) reported on SM delivered to the cervical spine (Table 2). Of these 12 studies, SM was delivered to humans in 9 (75.0%) studies and to inanimate objects (i.e. human analogue manikins: 2 (66.7%); load cell: 1 (33.3%)) in 3 (25.0%) studies. Ranges of reported force-time characteristics are reported in Table 3 (summary) and Appendix 3, Table A (full).

### Thoracic spine

Of the 66 included studies, 40 (60.6%) reported on SM delivered to the thoracic spine (Tables 4 and 5). Of these 40 studies, SM was delivered to humans in 27 (67.5%) studies and to inanimate objects (i.e. human analogue manikins: 12 (92.3%); strain gauge: 1 (7.7%)) in 13 (32.5%) studies. Ranges of reported force-time characteristics are reported in Table 3 (summary) and Appendix 3, Tables B-C (full).

**Table 4:** Summary of studies reporting on the force-time characteristics of spinal manipulation (SM) delivered to the thoracic spine of humans (n = 27)

Author/s Year, Country	SM delivery Profession (n)	Experience	Recipient/s (n)	Location/s	Technique/s	Interface/s	Measurement equipment	Metrological data
**Humans**								
Brennan et al. 1991, USA^6^	Clin Chiro (NR)	NR	Adult (80)	T1-6	PA	Pat-table	Force plate	No
Brennan et al. 1992, USA^7^	Clin Chiro (NR)	NR	Adult (6)	T2-6	HVLA	Pat-table	Force plate	No
Conway et al. 1993, Canada^14^	Clin Chiro (1)	NR	NR (10)	T4	UL hypothenar	Clin-pat	Pressure pad	No
Herzog et al. 1993, Canada^35^	Clin Chiro (60)	NR	NR (58)	T4	PA hypothenar	Clin-pat	Force pad	No
Gal et al. 1994, Canada^27^	Clin Chiro (1)	NR	Cadaver (2)	T11	Hypothenar	Clin-pat	Pressure pad	No
Cohen et al. 1995, USA^11^	Clin & Stud Chiro (30)	Clin: Mixed Stud: NR	NR (15)	T3-T10	BL transverse thenar	Pat-table	Force platform	No
Herzog et al. 1995, Canada^36^	Clin Chiro (1)	NR	Adult (2)	T3/T7/T9	PA hypothenar	Clin-pat	Force pad	No
Gal et al. 1997, Canada^28^	Clin Chiro (1)	NR	Cadaver (2)	T10-12	PA hypothenar	Clin-pat	Pressure pad	No
Kirstukas & Backman 1999, USA^43^	Clin Chiro (2)	> 5y	Adult (7)	T6-T9	UL thoracic	Clin-pat & Pat-table	Pressure sensor & Load cells	Yes
Herzog et al. 2001, Canada^37^	Clin Chiro (1)	< 5y	Adult (20)	T3-10	PA hypothenar	Clin-pat	Force pad	No
Van Zoest et al. 2003, England^65^	Clin Chiro (2)	> 5y	Adult (10)	T1-2/T4-5/ T8-9	Diversified	Clin-pat	Piezoelectric force sensor	Yes
Forand et al. 2004, Canada^25^	Clin Chiro (28)	Mixed	NR (9)	T4/T9	PA (clin choice)	Clin-pat	Sensor pad	No
Campbell & Snodgrass 2010, Australia^9^	Clin Physio (1)	> 5y	Adult (24)	T3-T10	Anterior AP	Pat-table	Load cells	Yes
Triano et al. 2011, Canada^62^	Clin & Stud Chiro (50)	Clin: >5y Y1: 102 h Y2: 218 h Y3: 326 h Y4: 409 h	NR (50)	Upper	Hypothenar transverse push	Pat-table	Force plate	Yes
Cambridge et al. 2012, Canada^8^	Clin Chiro (3)	> 5y	Adult (19)	T4-12	NR	Pat-table	Force plate	Yes
Gudavalli 2014, USA^32^	Clin Chiro (3)	NR	NR (5)	Thoracic	PA	Clin-pat	Force transducer	No
Williams & Cuesta-Vargas 2014, Spain^66^	Clin NR (2)	> 5y	Adult (13)	T5/6	PA	Clin-pat	Inertial sensor	No
Dunning et al. 2017, Italy^22^	Clin Physio (1)	> 5y	Adult (32)	T1/2	Lateral break	Skin mounted accelerometers	Accelerometer	No
Engell et al. 2019, Canada^24^	Clin Chiro (1)	> 5y	Adult (9)	T7	BL hypothenar push/Carver bridge	Clin-pat & Pat-table	Load cells & Force plate	No
Beyer et al. 2020, Belgium^5^	Clin & Stud NR (4)	Clin: NR Stud: NR (5th y)	Adult (16)	NR	AP	Pat-ground	Force plate	No
Dugailly et al. 2020, Belgium^21^	Clin & Stud NR (30)	Clin: NR Stud: NR	Adult (12)	NR	AP	Pat-ground	Force platform	No
Gorrell et al. 2020, Canada^29^	Clin Chiro (1)	> 5y	Adult (27)	T1/T4	PA	Clin-pat	Pressure pad	No
Joo et al. 2020, Korea^39^	Clin Physio (1)	NR	Adult (32)	T3/T7/T12	AP clenched fist/PA BL knife	Pat-table	Force plate	No
Pasquier et al. 2020, France^51^	Stud Chiro (136)	NR (3rd -6th y)	Adult (136)	NR	BL thenar push/modified pisiform	Pat-table	Force plate	Yes
Funabashi et al. 2021, Canada^26^	Clin Chiro (1)	> 5y	Geriatric (18)	T1-12	Clin choice	Clin-pat & Pat-table	Load cells & Force plate	Yes
Duarte et al. 2022, Canada^20^	Clin Chiro (1)	NR	Adult (19)	T6-9	PA	Pat-table	Force plate	Yes
Thomas et al. 2022, Canada^58^	Clin Chiro (1)	> 5y	Adult (40)	T7	Cross BL	Clin-pat & Pat-table	Load cells & Force plate	Yes

All superscript numbers in the first column refer to Appendix 2. Abbreviations: AP: anterior-posterior, BL: bilateral, Chiro: chiropractor, Clin: clinician, h: hours, HVLA: high velocity low amplitude, Mixed: experience of clinicians both > and < 5 years, (n): number of participants, NR: not reported, PA: posterior-anterior, Pat: patient, Physio: physiotherapist, SM: spinal manipulation, Stud: students, T: thoracic, UL: unilateral, Upper: upper thoracic spine, y: years, >: greater than, <: less than

**Table 5:** Summary of studies reporting on the force-time characteristics of spinal manipulation (SM) delivered to the thoracic spine of inanimate objects (e.g. human analogue manikins, instrumented tools) (n = 13)

Author/s Year, Country	SM delivery Profession (n)	Experience	Recipient/s (n)	Location/s	Technique/s	Interface/s	Measurement equipment	Metrological data
**Inanimate objects**								
Descarreaux et al. 2005, Canada^16^	Clin & Stud Chiro (43)	Clin:>5y Stud: NR (2nd /4th /final y)	Manikin (1)	NR	Hypothenar transverse	In Man & Clin-ground	Strain gauge & Force plate	No
Descarreaux et al. 2006, Canada^17^	Stud Chiro (31)	NR (4th y)	Manikin (1)	NR	Hypothenar transverse	In Man & Clin-ground	Load cell & Force plate	No
Descarreaux & Dugas 2010, Canada^18^	Stud Chiro (33)	NR (1st y)	Manikin (1)	NR	Hypothenar transverse	In Man & Clin-ground	Strain gauge & Force platform	No
Harvey et al. 2011, Canada/ USA^34^	Stud Chiro (87)	Clin: 330 h Stud: 330 h	Manikin (1)	NR	Hypothenar transverse	In Man & Clin-ground	Strain gauge & Force platform	No
Stemper et al. 2011, USA^56^	Clin Chiro (2)	> 5y	Manikin (1)	T7-T8	Clin choice	NR	Rotational potentiometer	No
Gudavalli 2014, USA^32^	Clin Chiro (3)	NR	Manikin (1)	Upper/ mid/ lower	PA	NR	NR	No
Triano et al. 2015, Canada^63^	Clin Chiro (38)	> 5y	Manikin (1)	T9	BL hypothenar/ BL thenar/cross-bilateral	Man-table	Force plate	Yes
Starmer et al. 2016, USA^55^	Stud Chiro (125)	NR (1st y)	Manikin (1)	T9	BL hypothenar/ BL thenar/cross-bilateral	Man-table	Force plate	No
Pasquier et al. 2017, France^49^	Stud Chiro (103)	NR (1st /3rd /5th y)	Strain gauge (1)	NR	PA transverse push	In Man	Strain gauge	No
Triano et al. 2017, Canada^64^	Clin Chiro (1)	> 5y	Manikin (1)	NR	BL-thumb/ ‘knife-edge’	Clin-Man & Man-table	Load cell & Force plate	Yes
Lardon et al. 2019, France^44^	Stud Chiro (113)	NR (1st y)	Manikin (1)	NR	PA	Man-table	Force plate	Yes
Pasquier et al. 2019, France^50^	Stud Chiro (137)	NR (4th /5th y)	Manikin (1)	NR	BL thenar push	Man-table	Force plate	Yes
Shannon et al. 2020, USA^54^	Clin & Stud Chiro (16)	Clin: Mixed Stud: NR (7–8/10trimesters)	Manikin (1)	T4	PA BL thenar	Man-table	Force plate	No

All superscript numbers in the first column refer to Appendix 2. Abbreviations: BL: bilateral, Chiro: chiropractor, Clin: clinician, h: hours, Lower: lower thoracic spine, Man: manikin, Mid: mid thoracic spine,Mixed: experience of clinicians both > and < 5 years, (n): number of participants, NR: not reported, PA: posterior-anterior, SM: spinal manipulation, Stud: students, Upper: upper thoracic spine, T: thoracic, y: years, >: greater than

### Lumbopelvic spine

Of the 66 included studies, 19 (28.8%) reported on SM delivered to the lumbopelvic spine (Table 6). Of these 19 studies, SM was delivered to humans in 12 (63.2%) studies and to inanimate objects (i.e. human analogue manikins: 5 (71.4%); force transducer: 1 (14.3%); both a rigid table-top and a human analogue manikin: 1 (14.3%)) in 7 (36.8%) studies. Ranges of reported force-time characteristics are reported in Table 3 (summary) and Appendix 3, Table D (full).

**Table 6:** Summary of studies reporting on the force-time characteristics of spinal manipulation (SM) delivered to the lumbopelvic spine of humans (n = 12) and inanimate objects (e.g. human analogue manikins, instrumented tools) (n = 7)

Author/s Year, Country	SM delivery Profession (n)	Experience	Recipient/s (n)	Location/s	Technique/s	Interface/s	Measurement equipment	Metrological data
**Humans**								
Hessell et al. 1990, Canada^38^	Clin Chiro (2)	NR	NR (6)	SIJ	Thompson technique	Clin-pat	Force pad	No
Herzog et al. 1993, Canada^35^	Clin Chiro (60)	NR	NR (58)	SIJ	PA drop-piece	Clin-pat	Force pad	No
Triano & Schultz 1997, USA^59^	Clin Chiro (6)	Mixed	Adult (11)	Lumbar/ SIJ	Mamillary push/ hypothenar ischial/ long lever lumbar	Pat-table	Force plate	No
Rogers & Triano 2003, USA^53^	Stud Chiro (16)	NR (2nd sem)	NR (16)	L5	Mamillary push	Pat-table	Force plate	No
Van Zoest & Gosselin 2003, England^65^	Clin Chiro (2)	> 5y	Adult (10)	SIJ	Diversified	Clin-pat	Force sensor	Yes
Triano et al. 2004, USA/Canada^60^	Clin & Stud Chiro (85)	Clin: >5y Stud: ~100 h	Adult (85)	L4	Mamillary push	Pat-table	Force plate	No
Triano et al. 2006, USA^61^	Stud Chiro (40)	NR (2nd y)	Adult (40)	L4	Mamillary push	Pat-table	Force plate	No
Gudavalli et al. 2013, USA^31^	Clin Chiro (3)	> 5y	Adult (5)	NR	Side posture	Clin-pat & Clin-ground	Force transducer & Force plate	No
Gudavalli 2014, USA^32^	Clin Chiro (3)	NR	NR (5)	Lumbar/ SIJ	Side-lying	Clin-pat	Force transducer	No
Gudavalli & Rowell 2014, USA^33^	Clin Chiro (2)	NR	Adult (5)	Lumbar/ SIJ	Side-lying	Clin-pat	Force transducer	No
Currie et al. 2016, USA^15^	Clin Chiro (2)	> 5y	Adult (17)	L3/SIJ	Hypothenar side-lying	Clin-pat & Pat-table	Force transducer & Force plate	No
Mourad et al. 2019, Italy^46^	Clin Physio (1)	> 5y	Adult (34)	L5/S1	Mamillary process body drop	Skin-mounted accelerometers	NA	No
**Inanimate objects**								
Adams et al. 1984, USA^1^	Clin Chiro (37)	Mixed	Rigid tabletop / Manikin (1)	Ilium/L5	Thompson technique/ single hand contact	Clin-table/ Clin-man	Force transducer	No
Adams & Wood 1984, USA^2^	Clin & Stud Chiro (74)	Clin: Mixed Stud: NR (8th quart)	Manikin (1)	Ilium/L5	Pisiform contact/ Thompson technique	Clin-man	Force transducer	No
Adams & Wood 1985, NR^3^	Clin & Stud Chiro (148)	Clin: Mixed Stud: NR (8th /10th / 12th quart)	Manikin (1)	PSIS/L5	Thomson technique	Clin-man	Force transducer	No
Gudavalli et al. 2013, USA^31^	Clin Chiro (2)	> 5y	Force transducer (1)	NR	HVLA	Clin-force transducer	Force transducer on force plate	No
Owens et al. 2016, USA^47^	Clin Chiro (11)	Mixed	Manikin (1)	PSIS/L5	Prone/side-posture Gonstead	Man-table	Force plate	No
Owens et al. 2017, USA^48^	Clin Chiro (11)	Mixed	Manikin (1)	L3	Reinforced pisiform Gonstead	Man-table	Force plate	No
Triano et al. 2017, Canada^64^	Clin Chiro (1)	> 5y	Manikin (2)	Lumbar/ SIJ	Prone-assisted/ lateral recumbent	Clin-man & Man-table	Load cell & Force plate	Yes

All superscript numbers in the first column refer to Appendix 2. Abbreviations: Chiro: chiropractor, Clin: clinician, h: hours, HVLA: high velocity low amplitude, L: lumbar, Man: manikin, Mixed: experience of clinicians both > and < 5 years, (n): number of participants, NR: not reported, PA: posterior-anterior, Pat: patient, Physio: physiotherapist, PSIS: posterior superior iliac spine, quart: quarter, sem: semester, SIJ: sacroiliac, SM: spinal manipulation, Stud: students, y: years, >: greater than

**Table 7:** Summary of studies reporting on the force-time characteristics of spinal manipulation (SM) delivered to inanimate objects (e.g. human analogue manikins, instrumented tools) with no region specified (n = 6)

Author/s Year, Country	SM delivery Profession (n)	Experience	Recipient/s (n)	Location/s	Technique/s	Interface/s	Measurement equipment	Metrological data
**Inanimate objects**								
McCarthy et al. 2002, England^45^	Clin Chiro (28)	Mixed	Tool (1)	L vertebra/tool	Superior-inferior	Clin-tool	Strain guage	No
Perle & Kawchuk 2005, Canada^52^	Clin Chiro (16)	> 5y	Rigid surface (1)	NR	Pisiform/hypothenar with or without arch in hand	Clin-tool	Pressure sensor	No
Kawchuk et al. 2006, Canada^42^	Clin Chiro (4)	NR	Force table/ plate (1)	Force mat	Hypothenar	Clin-tool	Load cell	No
Colloca et al. 2009, USA^12^	Clin Chiro (2)	NR	Tool (1)	NR	Toggle-torque-recoil	Clin-tool	Load cell	No
DeVocht et al. 2013, USA^19^	Clin & Stud Chiro (139)	Clin: NR Stud: NR	Tool (1)	Speeder board	Toggle recoil	Clin-tool	Force transducer	No
Colloca et al. 2020, UK^13^	Clin & Stud Chiro (53)	Clin: Mixed Stud: NR (3rd /5th y)	Force table/ plate (1)	NR	Toggle-torque-recoil	Clin-tool	Load cell	No

All superscript numbers in the first column refer to Appendix 2. Abbreviations: Chiro: chiropractor, Clin: clinician, L: lumbar, Mixed: experience of clinicians both > and < 5 years, (n): number of participants, NR: not reported, Stud: students, SM: spinal manipulation, y: years, >: greater than

### No region specified

Of the 66 included studies, 6 (9.1%) reported on SM delivered to a non-defined region (Table 7). Of these 6 studies, SM was delivered to a tool in 3 (50.0%) studies, a force table/plate in 2 (33.3%) studies and a rigid surface in 1 (16.7%) study. Ranges of reported force-time characteristics are reported in Table 3 (summary) and Appendix 3, Table E (full).

## Discussion

This review synthesised the current evidence describing force-time characteristics measured during the delivery of manual SM and highlights the considerable variability in these reported parameters. This finding is supported by an earlier systematic review by Downie and colleagues, the only review to date reporting on the force-time characteristics of SM delivered to all regions of the spine [11] and a recent critical literature review by Gyer and colleagues who investigated dose-response effects of the force-time characteristics of SM [12]. The current work is not directly comparable to these previous reviews due to methodological differences (i.e. systematic vs. critical vs. scoping review) and reporting differences (i.e. preload and peak force only vs. preload and peak force, rate of force application, time from end of preload to peak thrust force and thrust duration and data reported for different spinal regions). Additionally, the current search captured 35 additional studies reporting on force-time characteristics measured during SM since the publication of the 2010 systematic review and included considerably more studies reporting on the thoracic (n = 6 vs. n = 27) and lumbar (n = 7 vs. n = 12) spines than the 2022 critical review. This highlights that the current review has exhaustively included studies reporting on force-time characteristics of SM and that there has been a large increase in the number of publications reporting on SM force-time characteristic data in the past ~ 13 years.

Despite this increased reporting, the heterogeneity of the existing literature precluded synthesis of the reported data beyond descriptive analysis. Such heterogeneity included the following factors: (i) there were many SM ‘techniques’ used within and between spinal regions; (ii) SM was delivered by individuals with a wide range of clinical experience (e.g. novice student to experienced clinician); (iii) biomechanical data were collected at different locations (e.g. clinician-patient and patient-table interfaces); and (iv) using a variety of equipment (e.g. pressure sensor, load cells, force plates). Conceivably, this heterogeneity is one reason for the large variability in reported force-time characteristics of SM. While these differences in SM delivery likely reflect the rich tapestry of clinical practice in which treatment is tailored to individual patients chosen by the clinician delivering the intervention, such differences prevent between-study comparisons of results and subsequent statistical synthesis. This is one reason that informed the decision to conduct a scoping, rather than a systematic review. A scoping review allowed this heterogeneity to be captured and thus, this study reports exhaustively the range of force-time characteristics quantifying the delivery of SM. As such, this study provides a comprehensive summary of the force-time characteristics of SM delivered to both humans and inanimate objects.

There is a push within the literature for authors to quantify and report both passive (i.e. SM) [25] and active interventions (e.g. exercise) [26] in sufficient detail to allow for their replication in future research studies. The quantification and subsequent detailed reporting of interventions would facilitate: (i) accurate replication of the intervention in subsequent studies; (ii) improved interpretation of reported outcomes; and (iii) informed reader assessment regarding the applicability of both the intervention and reported outcomes to clinical practice [27]. However, it became evident during data extraction that detailed descriptions of several important items were not provided. Such items included vague or no reporting of: (i) the SM delivered; (ii) the individual who delivered the SM, especially their clinical training and experience; (iii) definitions of how each reported force-time characteristic was defined and/or calculated; (iv) information regarding the location of the applied SM; (v) the number of SM delivered; (vi) the number of SM recipients; and (vii) metrological details of the equipment used to quantify the force-time characteristics of SM.

### Recommendations for reporting of SM

For specific examples and suggested descriptions for the recommendations made below, readers are referred to the template for intervention description and replication (TIDieR) checklist (cited above) published by Hoffmann and colleagues in 2014 which provides a guide to be used by: (i) authors, to more easily structure the reporting of their interventions; (ii) reviewers and editors, to assess the descriptions; and (iii) readers, to determine the relevance of the reported results [27]. For examples of detailed reporting specific to SM (as discussed below), readers are referred to the previously cited consensus paper discussing guidelines for the reporting of spinal manipulative therapy interventions [25] and to a recent publication discussing the reporting of measurement equipment metrological details in reference to the quantification of force-time characteristics during SM and SMob [28]. To address the reporting deficiencies identified by the current review, the following general recommendations should be incorporated in future studies and their related publications. Firstly, there should be an adequate description of the applied SM technique. Regarding SM delivery, there should be sufficient detail so that an individual with manual therapy knowledge (e.g. manual therapy researchers, clinicians) would understand what was done and be able to replicate the intervention, including the primary direction of applied force (e.g. posterior-anterior), spinal region treated (e.g. thoracic) and level of treated segment (e.g. T3). Additional information regarding the location of the applied intervention (e.g. spinous process) should also be reported. The individual who delivered the SM should be described, including their training (e.g. physiotherapist, chiropractor) and experience delivering SM (e.g. students with X hours of experience delivering SM). Furthermore, the number of individuals delivering and receiving SM should be clearly reported, as should the number of SM that were delivered (i.e. how many thrusts were actually delivered). Secondly, definitions of how each reported force-time characteristic was defined and/or calculated need to be clearly reported. This allows for the comparison of data across multiple studies and will possibly facilitate meta-analysis of biomechanical data associated with dosage effects of SM in future clinical studies. Thirdly, as there is considerable variability in the reported force-time characteristics of SM, it is suggested that authors include raw data (i.e. non-analysed/non-averaged) to support their results where possible and that ranges are reported alongside other descriptive statistics (e.g. mean and standard deviation) for all reported force-time characteristics, allowing for a more illustrative description of the delivered SM. Finally, detailed description of the location of measurement (e.g. clinician-participant or participant-table) and measurement equipment used to quantify the force-time characteristics, including metrological details such as measurement error, reliability/repeatability, variability and calibration should be provided. With the use of appendices/supplementary files, it is feasible that these data are adequately reported while fulfilling editorial requirements (e.g. word limits).

### Limitations

Limitations of the current study include that only manuscripts published in English, French or German were included in the search strategy. Furthermore, as this was a scoping (and not systematic) review, it is possible that some manuscripts reporting on the force-time characteristics of SM were inadvertently not captured by the search strategy. However, every attempt was made to avoid this situation, with a broad search strategy inclusive of many professions that routinely use SM to treat patients with musculoskeletal disorders conducted across several databases, piloting and refinement of the search strategy prior to implementation, and the conduct of the scoping review in a systematic fashion (i.e. using two independent reviewers and data extractors). As such, it is unlikely that any seminal study was missed. Additionally, this review reports only on the kinetic force-time characteristics of SM and does not report on the kinematics of either the individual delivering the thrust and/or those of the recipient. Future reviews could address this gap in the literature by reporting on the kinematic parameters of individuals delivering SM (e.g. change in clinician centre of mass). Additionally, due to a lack of clarity regarding reported definitions of time to peak thrust force and thrust duration, it is possible that our best attempts to correctly classify this data were not sufficient. However, as two authors independently extracted the data prior to discussing and with a third author available for consensus resolution, it is unlikely that this lack of clarity in the original studies is a significant source of error within this study. Furthermore, it is not possible to determine the robustness/reliability of data collected using measurement equipment for which metrological data were not reported. Considering that this limitation applies to over three-quarters of the data reported here, the results should be interpreted with caution.

## Conclusion

Considerable variability in the reported kinetic force-time characteristics of SM exists. Some of this variability is likely due to differences in SM delivery and the measurement equipment used to quantify force-time characteristics. However, improved reporting in certain key areas could facilitate more sophisticated synthesises of force-time characteristics data in the future. Such syntheses could provide the foundation upon which dose-response estimates regarding the clinical effectiveness of SM are made.

### Electronic supplementary material

Below is the link to the electronic supplementary material.

Supplementary Material 1

Supplementary Material 2

Supplementary Material 3

## Data Availability

The datasets used and/or analysed during the current study are available from the corresponding author on reasonable request.

## List of abbreviations

3D: Three dimensional
CINAHL: Cumulative Index to Nursing and Allied Health Literature database
HVLA: High velocity, low amplitude
ICL: Index to Chiropractic Literature database
MEDLINE: Medical Literature Analysis and Retrieval System Online database
ms: Millisecond
N/s: Newtons per second
N: Newtons
n: Number of studies
PEDro: Physiotherapy Evidence database
PRISMA-ScR: Preferred Reporting Items for Scoping Reviews statement
s: Second
SM: Spinal manipulation
SMob: Spinal mobilization
SPIDER: Sample, Phenomenon of Interest, Design, Evaluation, Research Type search concept tool
US: United States
USA: United States of America
