# Supplementary material for: Spinal manipulation characteristics: a scoping literature review of force-time characteristics
Source: Chiropr Man Therap. 2023 Sep 13;31:36. doi: 10.1186/s12998-023-00512-1 (PMC10500795; doi:10.1186/s12998-023-00512-1)
Supplement: Supplementary file 1 — Supplementary Material 1 [file 12998_2023_512_MOESM1_ESM.docx]

# CINAHL search strategy

|  | Query | Limiters/expanders |
| --- | --- | --- |
| S1 | TI ((spine or spinal or medical) N3 (manip* or mobilisation* OR mobilization*)) OR (osteopath* or chiropract* or naprapath* or ((physiotherap* or TI ((manual or physical) N3 therap*)) and manip* OR mobilisation* OR mobilization*))) OR AB ((spine or spinal or medical) N3 (manip* or mobilisation* OR mobilization*)) OR (osteopath* or chiropract* or naprapath* or ((physiotherap* or AB ((manual or physical) N3 therap*)) and (manip* OR mobilisation* OR mobilization*))) | Expanders  - Apply equivalent subjects  Search modes  - Find all my search terms |
| S2 | (MH "Manipulation, Chiropractic") OR (MH "Manipulation, Osteopathic") | Expanders  - Apply equivalent subjects  Search modes  - Find all my search terms |
| S3 | S1 OR S2 | Expanders  - Apply equivalent subjects  Search modes  - Find all my search terms |
| S4 | TI (force* OR biomechanic* OR education* OR learning OR performance OR thrust OR dosage OR doseresponse OR kinetic OR kinematic) OR TI ((motor OR psychomotor OR man* OR  OMT OR mobilization OR mobilisation OR chiropract* OR practical OR osteopath* OR palpatory Or tactile OR practitioner OR therapist*) N6 skill*) OR AB (force* OR biomechanic* OR education* OR learning OR performance OR thrust OR dosage OR dose-response OR kinetic OR kinematic) OR AB ((motor OR  psychomotor OR man* OR OMT OR mobilization OR mobilisation OR chiropract* OR practical OR osteopath* OR palpatory or tactile OR practitioner OR therapist*) N6 skill*) | Expanders  - Apply equivalent subjects  Search modes  - Find all my search terms |
| S5 | TI (back OR neck OR spin* OR thoracic OR lumb* OR pelv* OR cervic* OR sacr* or joint) OR AB (back OR neck OR spin* OR thoracic OR lumb* OR pelv* OR cervic* OR sacr* or joint) | Expanders  - Apply equivalent subjects  Search modes  - Find all my search terms |
| S6 | S3 AND S4 AND S5 | Expanders  - Apply equivalent subjects  Search modes  - Find all my search terms |
| S7 | S3 AND S4 AND S5 | Limiters  - Language:  English, French, German  Expanders  - Apply equivalent subjects  Search modes  - Find all my search terms |
| S8 | S3 AND S4 AND S5 | Limiters  - Published Date:  20211001-; Language:  English, French, German  Expanders  - Apply equivalent subjects  Search modes  - Find all my search terms |

# ICL search strategy

|  | Query |
| --- | --- |
| S1 | All Fields:spinal manipulation OR All Fields:spinal mobilisation* OR All Fields:spinal mobilization* |
| S2 | All Fields:osteopath* OR All Fields:chiropract* OR All Fields:naprapath* OR All Fields:physiotherap* OR All Fields:\"manual therapy\" OR All Fields:\"physical therapy\" |
| S3 | All Fields:manip* OR All Fields:mobilisation* OR All Fields:mobilization* |
| S4 | All Fields:osteopath* OR All Fields:chiropract* OR All Fields:naprapath* OR All Fields:physiotherap* OR All Fields:\"manual therapy\" OR All Fields:\"physical therapy\" ANDAll Fields:manip* OR All Fields:mobilisation* OR All Fields:mobilization* |
| S5 | Subject:\"Manipulation, Chiropractic\" OR Subject:\"Manipulation, Osteopathic\" OR Subject:\"Manipulation, Spinal\" |
| S6 | All Fields:spinal manipulation OR All Fields:spinal mobilisation* OR All Fields:spinalmobilization* OR All Fields:osteopath* OR All Fields:chiropract* OR All Fields:naprapath* ORAll Fields:physiotherap* OR All Fields:\"manual therapy\" OR All Fields:\"physical therapy\" AND All Fields:manip* OR All Fields:mobilisation* OR All Fields:mobilization* ORSubject:\"Manipulation, Chiropractic\" OR Subject:\"Manipulation, Osteopathic\" ORSubject:\"Manipulation, Spinal\" |
| S7 | All Fields:force* OR All Fields:biomechanic* OR All Fields:education* OR All Fields:learning OR All Fields:performance OR All Fields:thrust OR All Fields:dosage OR All Fields:dose-response OR All Fields:kinetic OR All Fields:kinematic |
| S8 | All Fields:\"motor skills\" OR All Fields:\"psychomotor skills\" OR All Fields:\"manual skills\" OR All Fields:\"manipulation skills\" OR All Fields:OMT OR All Fields:\"mobilisation skills\" OR All Fields:\"mobilization skills\" OR All Fields:\"chiropractic skills\" OR All Fields:\"chiropractical skills\" OR All Fields:\"practical skills\" OR All Fields:\"ostheopathic skills\" OR All Fields:\"palpatory skills\" OR All Fields:\"tactile skills\" OR All Fields:\"practitioner skills\" OR All Fields:\"therapist skills\" |
| S9 | All Fields:force* OR All Fields:biomechanic* OR All Fields:education* OR All Fields:learning OR All Fields:performance OR All Fields:thrust OR All Fields:dosage OR All Fields:dose-response OR All Fields:kinetic OR All Fields:kinematic OR All Fields:\"motor skills\" OR AllFields:\"psychomotor skills\" OR All Fields:\"manual skills\" OR All Fields:\"manipulation skills\" OR All Fields:OMT OR All Fields:\"mobilisation skills\" OR All Fields:\"mobilization skills\" OR All Fields:\"chiropractic skills\" OR All Fields:\"chiropractical skills\" OR AllFields:\"practical skills\" OR All Fields:\"ostheopathic skills\" OR All Fields:\"palpatory skills\" OR All Fields:\"tactile skills\" OR All Fields:\"practitioner skills\" OR All Fields:\"therapist skills\" |
| S10 | All Fields:back OR All Fields:neck OR All Fields:spin* OR All Fields:thoracic OR All Fields:lumb* OR All Fields:pelv* OR All Fields:cervic* OR All Fields:sacr* OR All Fields:joint |
| S11 | All Fields:spinal manipulation OR All Fields:spinal mobilisation* OR All Fields:spinal mobilization* OR All Fields:osteopath* OR All Fields:chiropract* OR All Fields:naprapath* ORAll Fields:physiotherap* OR All Fields:\"manual therapy\" OR All Fields:\"physical therapy\" AND All Fields:manip* OR All Fields:mobilisation* OR All Fields:mobilization* OR Subject:\"Manipulation, Chiropractic\" OR Subject:\"Manipulation, Osteopathic\" ORSubject:\"Manipulation, Spinal\" AND All Fields:force* OR All Fields:biomechanic* OR AllFields:education* OR All Fields:learning OR All Fields:performance OR All Fields:thrust OR All Fields:dosage OR All Fields:dose-response OR All Fields:kinetic OR All Fields:kinematic OR All Fields:\"motor skills\" OR All Fields:\"psychomotor skills\" OR All Fields:\"manual skills\" OR All Fields:\"manipulation skills\" OR All Fields:OMT OR All Fields:\"mobilisation skills\" OR All Fields:\"mobilization skills\" OR All Fields:\"chiropractic skills\" OR All Fields:\"chiropractical skills\" OR All Fields:\"practical skills\" OR All Fields:\"ostheopathic skills\" OR All Fields:\"palpatory skills\" OR All Fields:\"tactile skills\" OR All Fields:\"practitioner skills\" OR All Fields:\"therapist skills\" AND All Fields:back OR All Fields:neck OR All Fields:spin* OR All Fields:thoracic OR All Fields:lumb* OR All Fields:pelv* OR All Fields:cervic* OR All Fields:sacr* OR All Fields:joint |

# Embase search strategy

|  | Query |
| --- | --- |
| #1 | (((spine OR spinal OR medical) NEAR/3 (manip* OR mobilisation* OR mobilization*)):ti,ab,kw) OR osteopath*:ti,ab,kw OR chiropract*:ti,ab,kw OR naprapath*:ti,ab,kw OR ((physiotherap*:ti,ab,kw OR (((manual OR physical) NEAR/3 therap*):ti,ab,kw)) AND (manip*:ti,ab,kw OR mobilisation*:ti,ab,kw OR mobilization*:ti,ab,kw)) |
| #2 | 'chiropractic manipulation'/de OR 'musculoskeletal manipulation'/de OR 'spine manipulation'/de OR 'osteopathic manipulation'/de |
| #3 | #1 OR #2 |
| #4 | force*:ti,ab,kw OR biomechanic*:ti,ab,kw OR education*:ti,ab,kw OR learning:ti,ab,kw OR performance:ti,ab,kw OR thrust:ti,ab,kw OR dosage:ti,ab,kw OR 'dose response':ti,ab,kw OR kinetic:ti,ab,kw OR kinematic:ti,ab,kw OR (((motor OR psychomotor OR man* OR omt OR mobilization OR mobilisation OR chiropract* OR practical OR osteopath* OR palpatory OR tactile OR practitioner OR therapist*) NEAR/6 skill*):ti,ab,kw) |
| #5 | back:ti,ab,kw OR neck:ti,ab,kw OR spin*:ti,ab,kw OR thoracic:ti,ab,kw OR lumb*:ti,ab,kw OR pelv*:ti,ab,kw OR cervic*:ti,ab,kw OR sacr*:ti,ab,kw OR joint:ti,ab,kw |
| #6 | #3 AND #4 AND #5 2331 |
| #7 | #3 AND #4 AND #5 AND [conference abstract]/lim |
| #8 | #3 AND #4 AND #5 NOT [conference abstract]/lim |
| #9 | #3 AND #4 AND #5 NOT [conference abstract]/lim AND ([english]/lim OR [french]/lim OR [german]/lim) |

# PEDro search strategy

|  | Search term combinations |
| --- | --- |
| S1 | "spinal manipulation" AND force*, "spinal manipulation" AND biomechanic*, "spinal manipulation" AND education*, "spinal manipulation" AND learning, "spinal manipulation" AND performance, "spinal manipulation" AND thrust, "spinal manipulation" AND dosage, "spinal manipulation" AND dose-response, "spinal manipulation" AND kinetic, "spinal manipulation" AND kinematic, "spinal manipulation" AND skills, "spinal mobilisation*" AND force*, "spinal mobilisation*" AND biomechanic*, "spinal mobilisation*" AND education*, "spinal mobilisation*" AND learning, "spinal mobilisation*" AND performance, "spinal mobilisation*" AND thrust, "spinal mobilisation*" AND dosage, "spinal mobilisation*" AND dose-response, "spinal mobilisation*" AND kinetic, "spinal mobilisation*" AND kinematic, "spinal mobilisation*" AND kinematic, "spinal mobilization*" AND force*, "spinal mobilization*" AND biomechanic*, "spinal mobilization*" AND education*, "spinal mobilization*" AND learning, "spinal mobilization*" AND performance, "spinal mobilization*" AND thrust, "spinal mobilization*" AND dosage, "spinal mobilization*" AND dose-response, "spinal mobilization*" AND kinetic, "spinal mobilization*" AND kinematic, "spinal mobilization*" AND skills |

# Cochrane Library search strategy

|  | Advanced search |
| --- | --- |
| #1 | ((spine or spinal or medical) NEAR/3 (manip* or mobilisation* OR mobilization*)):ti,ab,kw OR (osteopath* or chiropract* or naprapath* or ((physiotherap* or ((manual or physical) NEAR/3 therap*)) and (manip* OR mobilisation* OR mobilization*))):ti,ab,kw |
| #2 | (force* OR biomechanic* OR education* OR learning OR performance OR thrust OR dosage OR dose-response OR kinetic OR kinematic):ti,ab,kw OR ((motor OR psychomotor OR man* OR OMT OR mobilization OR mobilisation OR chiropract* OR practical OR osteopath* OR palpatory Or tactile OR practitioner OR therapist*) NEAR/6 skill*):ti,ab,kw |
| #3 | (back OR neck OR spin* OR thoracic OR lumb* OR pelv* OR cervic* OR sacr* or joint):ti,ab,kw |
| #4 | #1 AND #2 AND #3 |

# MEDLINE (Ovid) search strategy

|  | **Searches** |
| --- | --- |
| #1 | (((spine or spinal or medical) adj3 (manip* or mobilisation* or mobilization*)) or (osteopath* or chiropract* or naprapath* or ((physiotherap* or ((manual or physical) adj3 therap*)) and (manip* or mobilisation* or mobilization*)))).ti,ab,kw. |
| #2 | Manipulation, Chiropractic/ or Manipulation, Spinal/ or Musculoskeletal Manipulations/ or Manipulation, Osteopathic/ |
| #3 | 1 or 2 |
| #4 | (force* or biomechanic* or education* or learning or performance or thrust or dosage or dose-response or kinetic or kinematic or ((motor or psychomotor or man* or OMT or mobilization or mobilisation or chiropract* or practical or osteopath* or palpatory or tactile or practitioner or therapist*) adj6 skill*)).ti,ab,kw. |
| #5 | (back or neck or spin* or thoracic or lumb* or pelv* or cervic* or sacr* or joint).ti,ab,kw. |
| #6 | 3 and 4 and 5 |
| #7 | limit 6 to (english or french or german) |
