# Supplementary material for: Spinal manipulation characteristics: a scoping literature review of force-time characteristics
Source: Chiropr Man Therap. 2023 Sep 13;31:36. doi: 10.1186/s12998-023-00512-1 (PMC10500795; doi:10.1186/s12998-023-00512-1)
Supplement: Supplementary file 2 — Supplementary Material 2 [file 12998_2023_512_MOESM2_ESM.docx]

Appendix 2: Included studies reference list

1. Adams AA, Wood J. Forces used in selected chiropractic adjustments of the low back: a preliminary study. Res Forum. 1984 Autumn;1(1):5–9.

2. Adams AA, Wood J. Comparison of forces used in selected adjustments of the low back by exerienced chiropractors and chiropractic students with no clinical experience: a preliminary study. Res Forum. 1984 Autumn;1(1):16–23.

3. Adams AA, Wood J. Changes in force parameters with practice experience for selected low back adjustments. Res Forum. 1985 Winter;1(2):40–8.

4. Anderst WJ, Gale T, LeVasseur C, Raj S, Gongaware K, Schneider M. Intervertebral kinematics of the cervical spine before, during, and after high-velocity low-amplitude manipulation. Spine J. 12;18(12):2333–42.

5. Beyer B, Michaud A, Oliver T, Feipel V, Dugailly PM. Investigation of reaction force magnitude and orientation during supine thoracic thrust manipulation applied to intervertebral and costovertebral regions. Musculoskelet Sci Pract. 10;49:102217.

6. Brennan PC, Kokjohn K, Kaltinger CJ, Lohr GE, Glendening C, Hondras MA, et al. Enhanced phagocytic cell respiratory burst induced by spinal manipulation: potential role of substance P. J Manipulative Physiol Ther. 1991 Sep;14(7):399–408.

7. Brennan PC, Triano JJ, McGregor M, Kokjohn K, Hondras MA, Brennan DC. Enhanced neutrophil respiratory burst as a biological marker for manipulation forces: duration of the effect and association with substance P and tumor necrosis factor. J Manipulative Physiol Ther. 1992 Feb;15(2):83–9.

8. Cambridge ED, Triano JJ, Ross JK, Abbott MS. Comparison of force development strategies of spinal manipulation used for thoracic pain. Manual Ther. 2012 Jun;17(3):241–5.

9. Campbell BD, Snodgrass SJ. The effects of thoracic manipulation on posteroanterior spinal stiffness. J Orthop Sports Phys Ther. 2010 Nov;40(11):685–93.

10. Chang L, Wang H, Guo Y, Cai Z, Zhan H. Experimental and numerical analysis of biomechanical effects in cervical spine positioning rotation manipulation. Int J Numer Method Biomed Eng. 2022 Sep 27;e3651.

11. Cohen E, Triano JJ, McGregor M, Papakyriakou M. Biomechanical performance of spinal manipulation therapy by newly trained vs. practicing providers: does experience transfer to unfamiliar procedures? J Manipulative Physiol Ther. 1995 Jul;18(6):347–52.

12. Colloca CJ, Cunliffe C, Pinnock MH, Kim YK, Hinrichs RN. Force-time profile characterization of the McTimoney Toggle-Torque-Recoil Technique. J Manipulative Physiol Ther. 2009;32(5):372–8.

13. Colloca CJ, Cunliffe C, Hegazy MA, Pinnock M, Hinrichs RN. Measurement and analysis of biomechanical outcomes of chiropractic adjustment performance in chiropractic education and practice. J Manipulative Physiol Ther. 2020 Mar;43(3):212–24.

14. Conway PJ, Herzog W, Zhang Y, Hasler EM, Ladly K. Forces required to cause cavitation during spinal manipulation of the thoracic spine. Clin Biomech. 1993 Jul;8(4):210–4.

15. Currie SJ, Myers CA, Krishnamurthy A, Enebo BA, Davidson BS. Methods of muscle activation onset timing recorded during spinal manipulation. J Manipulative Physiol Ther. 5;39(4):279–87.

16. Descarreaux M, Dugas C, Raymond J, Normand MC. Kinetic analysis of expertise in spinal manipulative therapy using an instrumented manikin. J Chiropr Med. 2005;4(2):53–60.

17. Descarreaux M, Dugas C, Lalanne K, Vincelette M, Normand MC. Learning spinal manipulation: the importance of augmented feedback relating to various kinetic parameters. Spine J. 2006 Mar;6(2):138–45.

18. Descarreaux M, Dugas C. Learning spinal manipulation skills: assessment of biomechanical parameters in a 5-year longitudinal study. J Manipulative Physiol Ther. 2010 Mar;33(3):226–30.

19. DeVocht JW, Owens EF, Gudavalli MR, Strazewski J, Bhogal R, Xia T. Force-time profile differences in the delivery of simulated toggle-recoil spinal manipulation by students, instructors, and field doctors of chiropractic. J Manipulative Physiol Ther. 2013 Jul;36(6):342–8.

20. Duarte FCK, Funabashi M, Starmer D, Partata WA, West DWD, Kumbhare DA, et al. Effects of distinct force magnitude of spinal manipulative therapy on blood biomarkers of inflammation: a proof of principle study in healthy young adults. J Manipulative Physiol Ther. 2022;45(1):20–32.

21. Dugailly PM, Michaud A, Feipel V, Beyer B. Reaction force magnitude and orientation during supine thoracic spine thrust manipulation: an exploratory analysis and reliability of preload and impulse phase. J Manipulative Physiol Ther. 2020 Jul;43(6):597–605.

22. Dunning J, Mourad F, Zingoni A, Iorio R, Perreault T, Zacharko N, et al. Cavitation sounds during cervicothoracic spinal manipulation. Int J Sports Phys Ther. 2017 Aug;12(4):642–54.

23. Duquette SA, Starmer DJ, Plener JB, DAG BS. A pilot study to determine the consistency of peak forces during cervical spine manipulation utilizing mannequins. J Chiropractic Educ. 2021 Mar;35(1):8–13.

24. Engell S, Triano JJ, Howarth SJ. Force transmission between thoracic and cervical segments of the spine during prone-lying high-velocity low-amplitude spinal manipulation: A proof of principle for the concept of regional interdependence. Clin Biomech. 10;69:58–63.

25. Forand D, Drover J, Suleman Z, Symons B, Herzog W. The forces applied by female and male chiropractors during thoracic spinal manipulation. J Manipulative Physiol Ther. 2004 Jan;27(1):49–56.

26. Funabashi M, Son J, Pecora CG, Tran S, Lee J, Howarth SJ, et al. Characterization of thoracic spinal manipulation and mobilization forces in older adults. Clin Biomech. 2021 Aug 14;89:105450.

27. Gal J, Herzog W, Kawchuk G, Conway P, Zhang YT. Biomechanical studies of spinal manipulative therapy (SMT): quantifying the movements of vertebral bodies during SMT. J Can Chiropr Assoc. 1994 Mar;38(1):11–24.

28. Gal J, Herzog W, Kawchuk G, Conway PJ, Zhang YT. Movements of vertebrae during manipulative thrusts to unembalmed human cadavers. J Manipulative Physiol Ther. 1997 Jan;20(1):30–40.

29. Gorrell LM, Conway PJ, Herzog W. Differences in force-time parameters and electromyographic characteristics of two high-velocity, low-amplitude spinal manipulations following one another in quick succession. Chiropr Man Therap. 12 08;28(1):67.

30. Graham BA, Clausen P, Bolton PS. A descriptive study of the force and displacement profiles of the toggle-recoil spinal manipulative procedure (adjustment) as performed by chiropractors. Manual Ther. 2010 Feb;15(1):74–9.

31. Gudavalli MR, DeVocht J, Tayh A, Xia T. Effect of sampling rates on the quantification of forces, durations, and rates of loading of simulated side posture high-velocity, low-amplitude lumbar spine manipulation. J Manipulative Physiol Ther. 2013 Jun;36(5):261–6.

32. Gudavalli MR. Instantaneous rate of loading during manual high-velocity, low-amplitude spinal manipulations. J Manipulative Physiol Ther. 2014 Jun;37(5):294–9.

33. Gudavalli M, Rowell R. Three-dimensional chiropractor-patient contact loads during side posture lumbar spinal aanipulation: a pilot study. Chiropr Man Ther. 2014;22(1):1–13.

34. Harvey MP, Wynd S, Richardson L, Dugas C, Descarreaux M. Learning spinal manipulation: a comparison of two teaching models. J Chiropractic Educ. 2011;25(2):125–31.

35. Herzog W, Conway PJ, Kawchuk GN, Zhang Y, Hasler EM. Forces exerted during spinal manipulative therapy. Spine. 1993 Jul;18(9):1206–12.

36. Herzog W, Conway PJ, Zhang YT, Gal J, Guimaraes AC. Reflex responses associated with manipulative treatments on the thoracic spine: a pilot study. J Manipulative Physiol Ther. 1995 May;18(4):233–6.

37. Herzog W, Kats M, Symons B. The effective forces transmitted by high-speed, low-amplitude thoracic manipulation. Spine. 2001 Oct;26(19):2105–10.

38. Hessell BW, Herzog W, Conway PJW, McEwen MC. Experimental measurement of the force exerted during spinal manipulation using the Thompson technique. J Manipulative Physiol Ther. 1990;13(8):448–53.

39. Joo S, Kim J, Lee Y, Song C. The biomechanical analysis of magnitude and direction of force by different techniques of thoracic spinal manipulation. Biomed Res Int. 2020;2020:8928071.

40. Kawchuk GN, Herzog W, Hasler EM. Forces generated during spinal manipulative therapy of the cervical spine: a pilot study. J Manipulative Physiol Ther. 1992 Jun;15(5):275–8.

41. Kawchuk GN, Herzog W. Biomechanical characterization (fingerprinting) of five novel methods of cervical spine manipulation. J Manipulative Physiol Ther. 1993 Nov;16(9):573–7.

42. Kawchuk GN, Prasad NG, McLeod RC, Liddle T, Li T, Zhu Q. Variability of force magnitude and force duration in manual and instrument-based manipulation techniques. J Manipulative Physiol Ther. 2006;29(8):611–8.

43. Kirstukas SJ, Backman JA. Physician-applied contact pressure and table force response during unilateral thoracic manipulation. J Manipulative Physiol Ther. 1999 Jun;22(5):269–79.

44. Lardon A, Pasquier M, Audo Y, Barbier-Cazorla F, Descarreaux M. Effects of an 8-week physical exercise program on spinal manipulation biomechanical parameters in a group of 1st-year chiropractic students. J Chiropractic Educ. 2019 Oct;33(2):118–24.

45. McCarthy PW, Prudden M, Byfield D. An investigation of the manipulative parameters of postgraduate chiropractors. Eur J Chiropr. 2002;50(1):15–25.

46. Mourad F, Dunning J, Zingoni A, Iorio R, Butts R, Zacharko N, et al. Unilateral and multiple cavitation sounds during lumbosacral spinal manipulation. J Manipulative Physiol Ther. 2019;42(1):12–22.

47. Owens EF Jr, Hosek RS, Sullivan SG, Russell BS, Mullin LE, Dever LL. Establishing force and speed training targets for lumbar spine high-velocity, low-amplitude chiropractic adjustments. J Chiropractic Educ. 2016 Mar;30(1):7–13.

48. Owens EF Jr, Hosek RS, Mullin L, Dever L, Sullivan SGB, Russell BS. Thrust magnitudes, rates, and 3-dimensional directions delivered in simulated lumbar spine high-velocity, low-amplitude manipulation. J Manipulative Physiol Ther. 2017 Jul;40(6):411–9.

49. Pasquier M, Cheron C, Dugas C, Lardon A, Descarreaux M. The effect of augmented feedback and expertise on spinal manipulation skills: an experimental study. J Manipulative Physiol Ther. 2017 Jul;40(6):404–10.

50. Pasquier M, Barbier-Cazorla F, Audo Y, Descarreaux M, Lardon A. Learning spinal manipulation: gender and expertise differences in biomechanical parameters, accuracy, and variability. J Chiropractic Educ. 2019 Mar;33(1):1–7.

51. Pasquier M, Cheron C, Barbier G, Dugas C, Lardon A, Descarreaux M. Learning spinal manipulation: objective and subjective assessment of performance. J Manipulative Physiol Ther. 2020 Mar;43(3):189–96.

52. Perle SM, Kawchuk GN. Pressures generated during spinal manipulation and their association with hand anatomy. J Manipulative Physiol Ther. 2005;28(4):265.e1-265.e7.

53. Rogers CM, Triano JJ. Biomechanical measure validation for spinal manipulation in clinical settings. J Manipulative Physiol Ther. 2003 Nov;26(9):539–48.

54. Shannon ZK, Vining RD, Gudavalli MR, Boesch RJ. High-velocity, low-amplitude spinal manipulation training of prescribed forces and thrust duration: A pilot study. J Chiropractic Educ. 2020 Oct;34(2):107–15.

55. Starmer DJ, Guist BP, Tuff TR, Warren SC, Williams MG. Changes in manipulative peak force modulation and time to peak thrust among first-year chiropractic students following a 12-week detraining period. J Manipulative Physiol Ther. 5;39(4):311–7.

56. Stemper BD, Hallman JJ, Peterson BM. An experimental study of chest compression during chiropractic manipulation of the thoracic spine using an anthropomorphic test device. J Manipulative Physiol Ther. 2011 Jun;34(5):290–6.

57. Symons B, Wuest S, Leonard T, Herzog W. Biomechanical characterization of cervical spinal manipulation in living subjects and cadavers. J Electromyogr Kinesiol. 2012 Oct;22(5):747–51.

58. Thomas J, Murphy T, Tran S, Howarth SJ, Starmer D, Funabashi M. Characteristics of forces at the clinician-patient and patient-table interfaces during thoracic spinal manipulation in asymptomatic adults are consistent with deformable body models. J Appl Biomech. 02 01;38(1):39–46.

59. Triano J, Schultz AB. Loads transmitted during lumbosacral spinal manipulative therapy. Spine. 1997 Sep;22(17):1955–64.

60. Triano JJ, Bougie J, Rogers C, Scaringe J, Sorrels K, Skogsbergh D, et al. Procedural skills in spinal manipulation: do prerequisites matter? Spine J. 2004 Sep;4(5):557–63.

61. Triano JJ, Scaringe J, Bougie J, Rogers C. Effects of visual feedback on manipulation performance and patient ratings. J Manipulative Physiol Ther. 2006 Jun;29(5):378–85.

62. Triano JJ, Gissler T, Forgie M, Milwid D. Maturation in rate of high-velocity, low-amplitude force development. J Manipulative Physiol Ther. 2011 Mar;34(3):173–80.

63. Triano JJ, Giuliano D, Kanga I, Starmer D, Brazeau J, Screaton CE, et al. Consistency and malleability of manipulation performance in experienced clinicians: a pre-post experimental design. J Manipulative Physiol Ther. 2015 Jul;38(6):407–15.

64. Triano JJ, Lester S, Starmer D, Hewitt EG. Manipulation peak forces across spinal regions for children using mannequin simulators. J Manipulative Physiol Ther. 2017 Mar;40(3):139–46.

65. Van Zoest GGJM, Gosselin G. Three-dimensionality of direct contact forces in chiropractic spinal manipulative therapy. J Manipulative Physiol Ther. 2003;26(9):549–56.

66. Williams JM, Cuesta-Vargas A. Quantification of prone thoracic manipulation using inertial sensor-derived accelerations. J Manipulative Physiol Ther. 2014 May;37(4):230–5.
