# Supplementary material for: Spinal manipulation characteristics: a scoping literature review of force-time characteristics
Source: Chiropr Man Therap. 2023 Sep 13;31:36. doi: 10.1186/s12998-023-00512-1 (PMC10500795; doi:10.1186/s12998-023-00512-1)
Supplement: Supplementary file 3 — Supplementary Material 3 [file 12998_2023_512_MOESM3_ESM.docx]

# Appendix 3: Results tables by region

## Table A: Biomechanical parameters reported by studies in which spinal manipulation (SM) was delivered to the cervical spine of humans (n=9) and non-humans (e.g. human analogue manikins, instrumented tools) (n=3).

| Author(s)  Year, Country | Preload force (N)  Mean ± SD (range) | Peak force (N)  Mean ± SD (range) | Rate of force application (N/s)  Mean ± SD (range) | Time to peak (ms)  Mean ± SD (range) | Thrust duration (ms)  Mean ± SD (range) |
| --- | --- | --- | --- | --- | --- |
| **Humans** | | | | | |
| Kawchuk et al  1992, Canada^40^ | (0-11) | 117.7 ± 15.6  (99.0-140.0) | NR | 47.5 ± 14.7  (30.0-65.0) | 101.7 ± 14.7  (90.0-120.0) |
| Herzog et al  1993, Canada^35^ | 0 | 118  (99-140) | NR | 48  (30-65) | 102  (90-120) |
| Kawchuk et al  1993, Canada^41^ | Lat break: 39.5 ± 4.9^#^ Gonstead: 24.7 ± 6.5^#^ Toggle: 1.9 ± 1.9^#^ Rot: 29.1 ± 4.3^#^ | Lat break: 102.2^P^ ± 6.8^#^ Gonstead: 109.8^P^ ± 5.6^#^ Toggle: 117.6^P^ ± 6.4^#^ Rot: 40.5^P^ ± 4.5^#^ | Lat break: 1.2^Δ^  Gonstead: 1.2^Δ^  Toggle: 2.5^Δ^  Rotation: 0.5^Δ^ | Lat break: 86.7^P^ ± 3.4^#^ Gonstead: 91.9^P^ ± 4.5^#^ Toggle: 47.5^P^ ± 6.0^#^ Rot: 79.1^P^ ± 4.1^#^ | NR |
| Van Zoest et al  2003, England^65^ | L: 32 ± 10 R: 32 ± 7 | L: 108 ± 20 R:110 ± 12 | NR | L: 93 ± 24 R: 89 ± 16 | NR |
| Symons et al  2012, Canada^57^ | Live:  Asym: 77.4 ± 45  NP: 92.5 ± 61.6 Combined: 85.0 ± 56 Cadavers: 162.0 ± 45 | Live: Asym: 190.4 ± 70.7  NP: 190.1 ± 91.6  Combined: 190.3 ± 85  Cadavers: 283.9 ± 54 | NR | Live: Asym: 195 ± 100 NP: 155 ± 80 Combined: 175 ± 100 Cadavers: 120 ± 30 | NR |
| Anderst et al  2018, USA^4^ | 9.4 ± 3.1 | 65.6 ± 3.9 | 440.4 ± 57.6 | NR | 130 ± 10 |
| Gorrell et al  2020, Canada^29^ | NR | Upper – 1^st^; 2^nd^ thrust: Asym: 273; 407 Symp: 136 ± 29; 174 ± 34 Lower (1^st^; 2^nd^) Asym: 193 ± 74; 248 ± 112 Symp: 174 ± 37; 229 ± 62 | Upper – 1^st^; 2^nd^ thrust:  Asym: 1074; 1787 Symp: 601 ± 269; 895 ± 214 Lower (1^st^; 2^nd^) Asym: 638 ± 98; 895 ± 328 Symp: 912 ± 271; 1502 ± 731 | Upper – 1^st^; 2^nd^ thrust: Asym: 180; 160 Symp: 93 ± 54; 104 ± 32 Lower (1^st^; 2^nd^) Asym: 147 ± 12; 147 ± 12 Symp: 103 ± 43; 112 ± 34 | NR |
| Duquette et al  2021, Canada^23^ | CON:10.4 ± 9.8  Interv: 12.7 ± 10.1 | CON: 46.9 ± 27.5  Interv: 48.5 ± 23.4 | NR | NR | NR |
| Chang et al  2022, China^10^ | C5L^X^: 78.4 ± 40.3 C5R^X^: 64.2 ± 32.0 Chin L^X^: 31.8 ± 12.9 Chin R^X^: 32.7 ± 13.0 | C5L^X^: 147.8 ± 47.8 C5R^X^: 133.3 ± 45.0 Chin L^X^: 76.8 ± 29.0 Chin R^X^: 66.8 ± 28.2 | NR | NR | NR |
| **Non-humans** | | | | | |
| Graham et al  2010, Australia^30^ | (0.002–5) | 111.2 ± 48.8 (18.2-246.1) | NR | All: 67.5 ± 13.1 (20-100) Clin: 61.5 ± 9.1 (37.5-77.5)  Stud: 70.7 ± 13.9 (20-100) | NR |
| Triano et al  2017, Canada^64^ | NR | Neonate: 19.5 ± 5.0 Infant: 27.9 ± 7.8 Toddler: 30.5 ± 9.2 Young child: 60.4 ± 4.6 Mid child: 88.0 ± 3.0 Adolescent: 112.4 ± 27.1 | NR | NR | NR |
| Duquette et al  2021, Canada^23^ | CON: 22.9 ± 26.4  Interv: 18.8 ± 18.3 | CON: 123.00 ± 51.1  Interv: 125.9 ± 54.2 | NR | NR | NR |

Abbreviations: Asym: asymptomatic, C: cervical, Clin: clinician, CON: control, Interv: intervention, Lat: lateral, L: left, Lower: lower cervical spine, Mid: middle-aged child, N: Newtons, NP: neck pain, NR: not reported, ms: milliseconds, R: right, Rot: rotation, s: second, SD: standard deviation, Stud: student, Symp: symptomatic, Upper: upper cervical spine, #: SE, Ρ: normalized mean, Δ: N/ms, X: unclear if values are mean ± SD.

## Table B: Biomechanical parameters reported by studies in which spinal manipulation (SM) was delivered to the thoracic spine of humans (n=27).

| Author(s)  Year, Country | Preload force (N)  Mean ± SD (range) | Peak force (N)  Mean ± SD (range) | Rate of force application (N/s)  Mean ± SD (range) | Time to peak (ms)  Mean ± SD (range) | Thrust duration (ms)  Mean ± SD (range) |
| --- | --- | --- | --- | --- | --- |
| **Humans** | | | | | |
| Brennan et al  1991, USA^6^ | NR | 676 ± 55 | NR | 136 ± 20 | NR |
| Brennan et al  1992, USA^7^ | NR | 878 ± 99 (abstract) 873 ± 99 (results) | NR | 91 ± 20 | 310 ± 70 |
| Conway et al  1993, Canada^14^ | 145 ± 54 | 400 ± 118 | 2907 ± 1230 | 150 ± 77 | NR |
| Herzog et al  1993, Canada^35^ | 139 ± 46 | 399 ± 119 | NR | 150 ± 77 | NR |
| Gal et al  1994, Canada^27^ | Cadaver1: 68.9 ± 17.4 Cadaver2: 100.0 ± 32.5 | Cadaver1: 518.5 ± 70.0 Cadaver2: 534.2 ± 117.6 | NR | NR | NR |
| Cohen et al  1995, USA^11^ | Exper: 264 ± 83 Inexper: 213 ± 83 | Exper: 669 ± 186 Inexper: 600 ± 133 | Exper^D^: 8700 ± 3277 Inexper^D^: 7198 ± 2281 | Exper: 99 ± 19  Inexper: 103 ± 14 | NR |
| Herzog et al  1995, Canada^36^ | Fast: 145 Slow: 0 | Fast: 400 ± 118 Slow: 325 ± 62 | NR | NR | NR |
| Gal et al  1997, Canada^28^ | T10: 82 ± 14 T11: 82 ± 29 T12: 82 ± 13 | T10: 509 ± 45 T11: 525 ± 90 T12: 562 ± 22 | T10: 5900 ± 380 T11: 6900 ± 1500 T12: 7000 ± 1300 | NR | NR |
| Kirstukas & Backman  1999, USA^43^ | Pat-table: 310 ± 62 | Pat-table: 1044 ± 186 Clin-pat: 1213 ± 212 | Pat-table: 15592 ± 3187 | Pat-table: 96 ± 8 | NR |
| Herzog et al  2001, Canada^37^ | 23.8 ± 24.5 | 238.2 ± 45.9 | 1368 ± 327 | 160 ± 21 | NR |
| Van Zoest et al  2003, England^65^ | T1-2 L^R^:103 ± 33 T1-2 R^R^: 105 ± 31 T4-5 L^R^: 227 ± 30 T8-9 R^R^: 226 ± 31 | T1-2 L^R^: 237 ± 76 T1-2 R^R^: 263 ± 67 T4-5 L^R^: 561 ± 61 T8-9 R^R^: 518 ± 48 | NR | T1-2 L: 126 ± 21 T1-2 R: 149 ± 24 T4-5 L: 123 ± 21 T8-9 R: 140 ± 39 | NR |
| Forand et al  2004, Canada^25^ | M; F  T4-5: 137 ± 58; 138 ± 63 T8-11: 155 ± 6; 101 ± 34 | M; F  T4-5: 462 ± 194; 482 ± 130 T8-11: 431 ± 179; 473 ± 171 | M; F  T4-5^Δ^: 2.6 ± 1.3; 2.7 ± 1.1  T8-11^Δ^: 2.2 ± 1.3; 3.4 ± 1.8 | M; F  T4-5: 120 ± 28; 132 ± 29 T8-11: 130 ± 25; 117 ± 21 | NR |
| Campbell & Snodgrass  2010, Australia^9^ | Min: 41 ± 27 Max: 43 ± 23 | 342 ± 79 | 2929 ± 792 | NR | 120 ± 10 |
| Triano et al  2011, Canada^62^ | NR | Y1: 488 ± 148 Y2:442 ± 87 Y3: 397 ± 96 Y4:290 ± 74 DC: 306 ± 130 | Y1: 1769 ± 830 Y2: 1788 ± 750 Y3: 3183 ± 1023 Y4: 3990 ± 1220 DC: 4306 ± 1565 | NR | NR |
| Cambridge et al  2012, Canada^8^ | S1: 157.7 ± 31.8 S2: 179.7 ± 24.3 S3: 109.1 ± 17.2 | S1: 566.0 ± 105.0 S2: 625.4 ± 51.6 S3: 814.7 ± 69.0 | S1: 3244.7 ± 731.6 S2: 1835.6 ± 693.4 S3: 4191.3 ± 635.8 | S1: 130 ± 20 S2: 280 ± 110 S3: 170 ± 10 | NR |
| Gudavalli  2014, USA^32^ | 98.69 ± 68.94  (12.52-254.47) | 369.67 ± 93.54  (212.30-562.68) | 1476.91 ± 763.371  (415.97-3780.00) | 215.83 ± 113.98  (109.00-541.00) | 702.11 ± 321.27  (318.00-1330.00) |
| Williams & Cuesta-Vargas  2014, Spain^66^ | NR | NR | L; R: Clin 1^G^: -6.19 ± 1.38;  -6.69 ± 2.08 Clin 2^G^: -2.18 ± 1.16;  -4.65 ± 0.83 | L; R: Clin 1: 111 ± 20;  113 ± 20 Clin 2: 148 ± 50;  161 ± 40 | NR |
| Dunning et al  2017, Italy^22^ | NR | NR | NR | NR | 60.77  (28.25, 97.42^A^) |
| Engell et al  2019, Canada^24^ | Locked; unlocked  PA: 180.3 ± 22.2;  172.3 ± 20.7 IS: 70.2 ± 16.2; 65.8 ± 8.9 ML: -1.3 ± 16.2; -2.6 ± 15.4 | Locked; unlocked  PA: 476.6 ± 24.6;  447.5 ± 39.1 IS: 183.8 ± 36.5; 169.6 ± 29.9 ML: -0.4 ± 42.2; -0.3 ± 41.8 | NR | NR | NR |
| Beyer et al  2020, Belgium^5^ | Intervertebral F(z) total: 395.8 ± 95.0 GRF total: 399.0 ± 95.0 Costovertebral: F(z) total: 338.1 ± 114.2 GRF total: 342.9 ± 114.0 | Intervertebral F(z) total: 654.5 ± 98.5 GRF total: 658.4 ± 97.8 Costovertebral: F(z) total: 563.5 ± 144.2 GRF total: 566.8 ± 145.2 | NR | NR | NR |
| Dugailly et al  2020, Belgium^21^ | 399.0 ± 95.0^R^ | 658.4 ± 97.8^R^ | NR | NR | NR |
| Gorrell et al  2020, Canada^29^ | NR | 1^st^; 2^nd^ thrust: Asym: 368 ± 71; 464 ± 109 Symp: 490 ± 77; 573 ± 90 | 1^st^; 2^nd^ thrust:  Asym: 1732 ± 461;  2724 ± 587 Symp: 2749 ± 661;  3710 ± 815 | 1^st^; 2^nd^ thrust: Asym: 142 ± 19; 120 ± 10 Symp:108 ± 27; 111 ± 6 | NR |
| Joo et al  2020, Korea^39^ | PA:  T3^Z^: min 591.50 ± 72.78   max 653.41 ± 122.81 T7^Z^: min 599.65 ± 79.16   max 670.75 ± 125.19 T12^Z^:min 532.20 ± 93.01   max 568.76 ± 90.60 | PA:  T3^Z^: 458.71 ± 92.64 T7^Z^: 487.90 ± 71.98 T12^Z^: 432.30 ± 47.04 AP: T3^Z^: 735.57 ± 148.24 T7^Z^: 769.45 ± 140.50 T12^Z^: 719.33 ± 131.73 | NR | NR | NR |
| Pasquier et al  2020, France^51^ | 144.27 ± 62.07 | 439.13 ± 143.23 | NR | 128.31 ± 20.97 | NR |
| Funabashi et al  2021, Canada^26^ | Clin-pat: 260.0 ± 41.7^R^ Pat-table: 236.9 ± 47.2^R^ | Clin-pat: 470.1 ± 46.4^R^ Pat-table: 463.2 ± 57.0^R^ | Clin-pat:1644.5 ± 314.2^R^ Pat-table:1639.4 ± 238.1^R^ | Clin-pat:165.3 ± 28.2^R^ Pat-table:169.8 ± 28.6^R^ | NR |
| Duarte et al  2022, Canada^20^ | CON: 195.4 ± 12.8 400N: 183.4 ± 13.8 800N: 197.8 ± 9.8 | CON: NA 400N: 393.4 ± 34.1 800N: 847.4 ± 95.1 | NR | CON: NA 400N: 101.1 ± 2.3 800N: 134.0 ± 14.8 | NR |
| Thomas et al  2022, Canada^58^ | Clin-pat: 272.7 ± 55.3^R^  Pat-table: 216.9 ± 30.1^R^ | Clin-pat: 778.4 ± 182.3^R^  Pat-table: 769.6 ± 64.0^R^ | Clin-pat:3109.1 ± 1397.0^R^ Pat-table:3677.7 ± 1119.5^R^ | Clin-pat:181.3 ± 44.9^R^  Pat-table:165.3 ± 44.0^R^ | NR |

Abbreviations: AP: anterior-posterior, Asym: asymptomatic, Clin: clinician, CON: control, DC: doctor of chiropractic, Exper: experience, F: female, GRF: ground reaction force, Inexper: inexperienced, IS: inferior-superior, L: left, M: male, Max: maximum, Min: minimum, ML: medial-lateral, ms: milliseconds, N: Newtons, NA: not applicable, NR: not reported, PA: posterior-anterior, Pat: patient, R: right, s: second, S: strategy, SD: standard deviation, Symp: symptomatic, T: thoracic, y: year, D: upper rise rate, R: F_resultant_, Δ: N/ms, G: m/s^2^, A: 95% CI, Z: Z-axis, F_(Z)_: forces in the main line of drive.

## Table C: Biomechanical parameters reported by studies in which spinal manipulation (SM) was delivered to the thoracic spine of non-humans (e.g. human analogue manikins, instrumented tools) (n=13).

| Author(s)  Year, Country | Preload force (N)  Mean ± SD (range) | Peak force (N)  Mean ± SD (range) | Rate of force application (N/s)  Mean ± SD (range) | Time to peak (ms)  Mean ± SD (range) | Thrust duration (ms)  Mean ± SD (range) |
| --- | --- | --- | --- | --- | --- |
| **Non-humans** | | | | | |
| Descarreaux et al  2005, Canada^16^ | G1: 31 ± 20 G2: 41 ± 19 G3: 77 ± 21 G4: 57 ± 21 | G1: 570 ± 27 G2: 569 ± 26 G3: 594 ± 29 G4: 544 ± 29 | G1: 3485 ± 279 G2: 3684 ± 267 G3: 4487 ± 292 G4: 4217 ± 292 | G1: 171 ± 10 G2: 159 ± 10 G3: 136 ± 11 G4: 140 ± 11 | NR |
| Descarreaux et al  2006, Canada^17^ | Base; Post FB: 66.1 ± 16.2^#^; 176.7 ± 17.3^#^ Standard: 94.9 ± 15.7^#^;  90.9 ± 16.8^#^ | Base; Post FB: 630.9 ± 35.0^#^; 538.4 ± 16.9^#^ Standard: 659.7 ± 33.8^#^;  603.4 ± 16.3^#^ | NR | Base; Post FB: 155 ± 13^#^; 147 ± 16^#^ Standard: 186 ± 16^#^;  203 ± 17^#^ | NR |
| Descarreaux & Dugas  2010, Canada^18^ | Y1: 88.0 ± 2.9^#^ Y2: 32.8 ± 4.8^#^ Y3: 124.1 ± 9.7^#^ Y4: 131.1 ± 8.2^#^ Y5: 116.1 ± 5.3^#^ Expert: 57 ± 21^#^ | Y1: 404.4 ± 8.6^#^ Y2: 460.2 ± 14.9^#^ Y3: 508.8 ± 16.5^#^ Y4: 497.9 ± 10.3^#^ Y5: 450.0 ± 14.4^#^ Expert: 544 ± 29^#^ | Y1: 2557 ± 249^#^ Y2: 3176 ± 214^#^ Y3: 4164 ± 222^#^ Y4: 4271 ± 149^#^ Y5: 3859 ± 181^#^ Expert: 4217 ± 292^#^ | Y1: 266 ± 46^#^ Y2: 168 ± 10^#^ Y3: 134 ± 6^#^ Y4: 119 ± 4^#^ Y5: 122 ± 4^#^ Expert: 140 ± 11^#^ | NR |
| Harvey et al  2011, Canada/USA^34^ | G1: 131.5 ± 21.6 G2: 155.3 ± 55.6 | G1: 425.9 ± 67.5 G2: 481.2 ± 58.3 | G1: 2960.4 ± 1016.8 G2: 3840.3 ± 1496.9 | G1: 112 ± 25 G2: 101 ± 35 | NR |
| Stemper et al  2011, USA^56^ | NR | 429 ± 289 | NR | NR | NR |
| Gudavalli  2014, USA^32^ | 98.69 ± 68.94  (12.52-254.47) | 369.67 ± 93.54  (212.30-562.68) | 1476.91 ± 7633.71  (415.97-3780.00) | 215.83 ± 113.98  (109.00-541.00) | 702.11 ± 321.27  (318.00-1330.00) |
| Triano et al  2015, Canada^63^ | Cross-BL: 154.1 ± 78.1 BL thenar: 145.7 ± 59.4 BL hypothenar: 171.7 ± 58.4 | Cross-BL: 392.6 ± 155.5 BL thenar: 401.1 ± 127.5 BL hypothenar: 336.5 ± 92.7 | Cross-BL: 3207.7 ± 1479.2 BL thenar: 3126.3 ± 1008.5 BL hypothenar: 3275.2 ± 1214.5 | NR | NR |
| Starmer et al  2016, USA^55^ | NR | NR | NR | 600N: Pre: 136.835 ± 40.48  Post: 125.385 ± 33.78 400N: Pre: 137.094 ± 42.47 Post: 125.385 ± 37.46 | NR |
| Pasquier et al  2017, France^49^ | G1; G2; G3 Base: 104.5 (91.9-116.9^A^); 118.7 (106.4-131^A^); 107.6 (94.5-120.6^A^)  Post: 120.6 (111.1-130.1^A^); 129.5 (120-138.9^A^); 115.6 (105.7-125.6^A^)  Retention: 132.5 (121.8-143.2^A^); 128.3 (117.7-138.9^A^); 118.0 (106.8-129.2^A^) | G1; G2; G3 Base: 289.5 (273-306^A^); 301.6 (285-318^A^); 286.5 (269-303^A^)  Post: 291.7 (280-303^A^); 303.7 (292-315^A^); 288.8 (276-300^A^)  Retention: 293.0 (281-304^A^); 287.6 (276-298^A^); 292.4 (277-301^A^) | G1; G2; G3 Base: 1963.1 (1700-2225^A^); 2306.3 (2047-2564^A^); 2495.2 (2221-2769^A^)  Post: 1648.3 (1436-1859^A^); 2020.6 (1812-2229^A^); 2102.9 (1881-2324^A^)  Retention: 1472.5 (1257-1688^A^); 1736.6 (1524-1948^A^); 2022.8 (1797-2248^A^) | G1; G2; G3 Base: 132 (117-151^A^); 97 (79-116^A^); 82 (62-101^A^)  Post: 125 (112-139^A^); 101 (87-114^A^); 100 (86-114^A^)  Retention: 130 (119-142^A^); 102 (91-113^A^); 98 (86-109^A^) | NR |
| Triano et al  2017, Canada^64^ | NR | Neonate:17.4 ± 1.2 Infant:19.9 ± 1.3 Toddler1: 34.8 ± 4.3 Toddler2: 117.8 ± 6.1 Young child: 209.8 ± 16.9 Mid child: 243.0 ± 19.7 Adolescent: 393.4 ± 40.8 | NR | NR | NR |
| Lardon et al  2019, France^44^ | NR | NR | NR | NR | 1^st^; 2^nd^ session 125.1 ± 19.3 (87.4-196.3); 126.0 ± 15.7 (89.4-176.6) |
| Pasquier et al  2019, France^50^ | F; M 4^th^: 138.61 ± 47.94;  149.30 ± 57.71 5^th^: 136.98 ± 45.72;  165.225 ± 59.72 | F; M 4^th^: 467.65 ± 79.45;  536.27 ± 144.44 5^th^: 431.79 ± 66.95;  504.27 ± 78.63 | F; M 4^th^: 2699.56 ± 543.59;  3489.76 ± 1012.66 5^th^: 2381.49 ± 495.72;  3151.89 ± 729.36 | F; M 4^th^: 123 ± 13;  113 ± 20 5^th^: 126 ± 20;  109 ± 13 | NR |
| Shannon et al  2020, USA^54^ | NR | NR | NR | NR | 350 N: Stud; DC Base^*^: 113 (101–139);  132 (93–138) Post^*^: 106 (105–137);  158 (126–198) 1w post^*^: 112 (99–134);  135 (103–192) 4w post^*^: 122 (108–124); 125 (102–163) 8w post^*^: 118 (105–140); 132 (95–161) 550 N: Stud; DC Base^*^: 115 (105–133);  135 (106–160) Post^*^: 107 (105–113);  152 (130–171) 1w post^*^: 109 (97–113);  158 (105–175) 4w post^*^: 101 (96–116);  140 (106–166) 8w post^*^: 109 (100–136); 140 (116–160) |

Abbreviations: Base: baseline, BL: bilateral, DC: Doctor of Chiropractic, Exper: experience, F: female, FB: feedback, G: group, M: male, ms: milliseconds, N: Newton, NR: not reported, Post: post-intervention, SD: standard deviation, s: seconds, Stud: students, y: year, w: week, #: SE, A: 95% CI, *: median (IQR).

## Table D: Biomechanical parameters reported by studies in which spinal manipulation (SM) was delivered to the lumbopelvic spine of humans (n=12) and non-humans (e.g. human analogue manikins, instrumented tools) (n=7).

| Author(s)  Year, Country | Preload force (N)  Mean ± SD (range) | Peak force  Mean ± SD (range) | Rate of force application (N/s)  Mean ± SD (range) | Time to peak (ms)  Mean ± SD (range) | Thrust duration (ms)  Mean ± SD (range) |
| --- | --- | --- | --- | --- | --- |
| **Humans** | | | | | |
| Hessell et al  1990, Canada^38^ | (20-180) | (220-550) | NR | NR | (200-420) |
| Herzog et al  1993, Canada^35^ | 88 ± 78 | 328 ± 78 | NR | NR | NR |
| Triano & Schultz  1997, USA^59^ | NR | MP: 495.5 ± 142.5^A^ HI: 515.5 ± 123.8^A^ LL: 384.7 ± 114.1^A^ | MP: 2176.6  HI: 2483.4  LL: 1806.7 | NR | NR |
| Rogers & Triano  2003, USA^53^ | NR | Expert: 375.5 Time 1: 127.88 Time 2: 178.27 | Expert: 1649.53 Time 1: 630 Time 2: 817.6 | NR | NR |
| Van Zoest & Gosselin  2003, England^65^ | L^R^: 66 ± 17 R^R^: 83 ± 15 | L^R^: 237 ± 95 R^R^: 241 ± 57 | NR | L: 182 ± 42 R: 166 ± 35 | NR |
| Triano et al  2004, USA/Canada^60^ | NR | Prog1: 210.2 ± 106.5^A^ Prog2: 321.4 ± 112.6^A^ Expert: 488.3 ± 125.7^A^ | Prog1: 840.7 ± 148.8^A^ Prog2: 2223.1 ± 384.2^A^ Expert: 3812.6 | NR | Expert: 320 |
| Triano et al  2006, USA^61^ | NR | Base – CON; visual FB: 315.6; 312.0 Post FB – G2: 372 | Base – CON; visual FB: 2167.6; 2132.0 Post FB – G2: 2761 | NR | Base – CON; visual FB: 440; 410 Post FB – G2: 330 |
| Gudavalli et al  2013, USA^31^ | 106.3 ± 55.5 | 327.6 ± 90.7 | 1077.6 ± 571.9.2 | 261 ± 163 | 770 ± 212 |
| Gudavalli  2014, USA^32^ | 101.57 ± 54.31  (46.47-190.13) | 336.08 ± 87.73  (105.84-441.11) | 972.86 ± 432.27  (202.11-1621.00) | 281.14 ± 193.06  (195.00-938.00) | 997.92 ± 239.60  (727.00-1557.00) |
| Gudavalli & Rowell  2014, USA^33^ | 98.7 ± 29.3^R^ | 340.3 ± 75.2^R^ | 1595.1 ± 749.8 | 164 ± 37 | 2876 ± 1240 |
| Currie et al  2016, USA^15^ | NR | 529.5 ± 152.4  (242.2-940.2) | NR | 243 ± 80 | NR |
| Mourad et al  2019, Italy^46^ | NR | NR | NR | NR | 139.13  (5.61-493.79^A^) |
| **Non-humans** | | | | | |
| Adams et al  1984, USA^1^ | NR | RI^Ø^: 56.6 ± 16.6  LI^Ø^: 55.8 ± 18.1  RLum^Ø^: 41.0 ± 15.0  LLum^Ø^: 42.1 ± 15.4 | RI^Κ^: 10.49 ± 6.38  LI^Κ^: 10.52 ± 6.73  RLum^Κ^: 9.25 ± 5.44  LLum^Κ^: 8.88 ± 6.16 | NR | RI: 371 ± 211 LI: 401 ± 219 RLum: 434 ± 249 LLum: 441 ± 276 |
| Adams & Wood  1984, USA^2^ | NR | RI^Ø^: 48.2 ± 14.0 LI^Ø^: 46.2 ± 11.9  RLum^Ø^: 32.38 ± 9.57  LLum^Ø^: 32.1 ± 9.01 | RI^Κ^: 7.68 ± 4.44 LI^Κ^: 7.84 ± 4.23  RLum^Κ^: 5.85 ± 4.26  LLum^Κ^: 5.82 ± 3.96 | NR | RI: 337 ± 176 LI: 348 ± 147 RLum: 357 ± 182 LLum: 348 ± 155 |
| Adams & Wood  1985, Unclear^3^ | NR | NR | NR | NR | Pelvis – R; L: 8^th^ quart Stud: 336.8 ± 175.7; 347.8 ± 147.2 10^th^ quart Stud: 497.0 ± 268.4; 488.9 ± 271.8 12^th^ quart Stud: 542.7 ± 266.9; 471.4 ± 255.5 DC: 371.1 ± 211.4; 400.5 ± 219.1  Lum – R; L:  8th quart Stud: 259.1 ± 176.3; 260.3 ± 189.3 10^th^ quart Stud: 356.4 ± 232.2; 359.1 ± 263.5 12^th^ quart Stud: 389.1 ± 240.1; 427.1 ± 283.6 DC: 41.14 ± 242.1; 395.0 ± 273.9 |
| Gudavalli et al  2013, USA^31^ | 94.4 ± 71.7 | 432.5 ±122.8 | 2692 ± 1633 | 154 ± 70 | 574 ± 260 |
| Owens et al  2016, USA^47^ | Prone:  Light: 138.4 ± 78.2  Normal: 161.0 ± 76.6  Heavy: 163.4 ± 87.3  Side-posture:  Light: 94.7 ± 62.9  Normal: 111.3 ± 62.8  Heavy: 103.1 ± 66.8 | Prone: Light: 379.3 ± 173.4  Normal: 475.1 ± 169.4  Heavy: 694.0 ± 291.3  Side-posture:  Light: 356.8 ± 150.1  Normal: 433.6 ± 155.7  Heavy: 580.3 ± 202.4 | Prone:  Light: 2800 ± 1790  Normal: 2940 ± 1190  Heavy: 4610 ± 2620  Side-posture:  Light: 2450 ± 1650  Normal: 2880 ± 1490  Heavy: 4640 ± 2070 | NR | NR |
| Owens et al  2017, USA^48^ | P:  Light: 121.2 ± 72.4 Normal: 141.4 ± 76.6 Heavy: 141.5 ± 80.1 PLS:  Light: 122.1 ± 85.9 Normal: 155.4 ± 83.7 Heavy: 157.7 ± 95.5 PRS:  Light: 144.0 ± 75.6 Normal: 157.0 ± 74.4 Heavy: 161.8 ± 90.9 | P:  Light: 323.9 ± 129.6 Normal: 431.7 ± 179.5 Heavy: 644.8 ± 285.4 PLS:  Light: 357.2 ± 130.2 Normal: 476.4 ± 165.6 Heavy: 687.0 ± 291.7 PRS:  Light: 412.9 ± 270.3 Normal: 453.9 ± 158.8 Heavy: 713.6 ± 319.8 | P:  Light^Δ^: 2.6 ± 1.2  Normal^Δ^: 2.8 ± 1.2  Heavy^Δ^: 4.5 ± 2.9  PLS:  Light^Δ^: 2.7 ± 1.1  Normal^Δ^: 3.0 ± 1.2  Heavy^Δ^: 4.9 ± 2.3  PRS:  Light^Δ^: 3.4 ± 3.3  Normal^Δ^: 3.1 ± 1.1  Heavy^Δ^: 5.0 ± 3.5 | P:  Light: 141.8 ± 73.9 Normal: 155.6 ± 57.9 Heavy: 157.5 ± 56.4 PLS:  Light: 153.8 ± 91.6 Normal: 175.5 ± 107.0 Heavy: 155.8 ± 58.1 PRS:  Light: 148.9 ± 81.2 Normal: 160.8 ± 87.5 Heavy: 161.2 ± 76.1 | NR |
| Triano et al  2017, Canada^64^ | NR | Lum: Neonate: 17.6 ± 2.1 Infant: 21.9 ± 3.7 Toddler: 89.5 ± 7.2 Young child: 175.0 ± 26.1 Mid child: 296.9 ± 20.2 Adolescent: 387.2 ± 43.1  SIJ: Neonate: 52.3 ± 10.0 Infant: 84.9 ± 15.2 Toddler: 101.8 ± 5.3 Young child: 222.7 ± 20.7 Mid child: 369.9 ± 23.0 Adolescent: 561.2 ± 33.9 | NR | NR | NR |

Abbreviations: Base: baseline, CON: control, DC: Doctor of Chiropractic, FB: feedback, G: group, HI: hypothenar ischial, I: ilium, L: left, LL: long lever lumbar, Lum: lumbar, MP: mamillary push, ms: milliseconds, N: Newtons, NR: not reported, P/PLS/PRS: Gonstead listing, Prog: program, quart: quarter, R: right, s: second, SD: standard deviation, SIJ: sacroiliac, Stud: students, A: 95% CI (as reported by authors), R: F_resultant_, Ø: pounds, Κ: pounds/s, Δ: N/ms.

## Table E: Biomechanical parameters reported by studies in which spinal manipulation (SM) was delivered to non-humans (e.g. human analogue manikins, instrumented tools) with no region specified (n=6).

| Author(s)  Year, Country | Preload force (N)  Mean ± SD (range) | Peak force (N)  Mean ± SD (range) | Rate of force application (N/s)  Mean ± SD (range) | Time to peak (ms)  Mean ± SD (range) | Thrust duration (ms)  Mean ± SD (range) |
| --- | --- | --- | --- | --- | --- |
| **Non-humans** | | | | | |
| McCarthy et al  2002, England^45^ | NR | NR | 1-5y ^Δ^: 708 ± 87  (603-865)  6-10y ^Δ^: 931 ± 233  (551-1303)  11-14y ^Δ^: 1082 ± 209  (838-1297)  22-24y ^Δ^: 1260 ± 436  (854-1692) | 1-5y: 98 ± 23.1  (74.7-136.7) 6-10y: 71.83 ± 18.95  (43.3-108) 11-14y: 64 ± 7.38  (52-70) 22-24y: 58.75 ± 8.11  (48.7-67) | NR |
| Perle & Kawchuk  2005, Canada^52^ | NR | Arched^#Σ^: 191.38 ± 16.56  Flat ^# Σ^: 278.06 ± 21.870 | NR | NR | NR |
| Kawchuk et al  2006, Canada^42^ | NR | E1: 253.654 ± 37.738 E2: 157.408 ± 24.624 Nov1: 256.773 ± 49.836 Nov2: 387.432 ± 61.526 | NR | E1: 66.924 ± 5.152 E2: 99.882 ± 13.897 Nov1: 164.228 ± 55.745 Nov2: 250.889 ± 87.429 | NR |
| Colloca et al  2009, USA^12^ | NR | 87.22 ± 24.18 | NR | 12.31 ± 4.39 | 36.38 ± 9.58 |
| DeVocht et al  2013, USA^19^ | I: 53.7 (9.2) G1; G2 Sess1*: 65.7 (53.3, 71.5); 64.9 (52.8, 76.7) Sess2*: 67.4 (61.6, 74.4); 70.4 (62.5, 76.9) Sess3*: 62.1 (58.7, 67.8); 63.1 (58.9, 70.4) DC*: 66.7 (57.8, 73.1) | NR | I ^Δ^: 2.03 (0.77)  G1; G2 Sess1*^Δ^: 2.32 (1.81, 3.24); 2.67 (1.81, 3.44)  Sess2*^Δ^: 2.26 (1.78, 3.23); 2.57 (2.15, 3.60) Sess3*^Δ^: 2.02 (1.67, 2.82); 2.13 (1.74, 2.96)  DC ^Δ^*: 5.00 (3.04, 6.94) | NR | I: 75.4 (18.5) G1; G2 Sess1*: 77.8 (70.8, 98.1); 73.8 (66.6, 85.1)  Sess2*: 79.7 (68.3, 97.0); 76.2 (64.4, 94.0)  Sess3*: 77.3 (66.7, 94.0); 72.0 (64.8, 88.0)  DC*: 47.8 (38.5, 69.1) |
| Colloca et al  2020, UK^13^ | 8.64 ± 8.79 | 45.84 ± 28.62 | NR | 134.67 ± 202.58 | 180.56 ± 201.73 |

Abbreviations: Arched: arched hand configuration, DC: Doctor of Chiropractic, E: expert, Flat: flat hand configuration, G: group, I: instructors, N: Newtons, Nov: novice, NR: not reported, ms: milliseconds, SD: standard deviation, s: second, Sess: student session, y: year; Δ: N/ms, #: SE, Σ: N/cm^2^, *: median (IQR).
